# A trifunctional peptide broadly inhibits SARS-CoV-2 Delta and Omicron variants in hamsters

**DOI:** 10.1038/s41421-022-00428-9

**Published:** 2022-06-30

**Authors:** Hanjun Zhao, Kelvin Kai-Wang To, Hoiyan Lam, Chuyuan Zhang, Zheng Peng, Xinjie Meng, Xiankun Wang, Anna Jinxia Zhang, Bingpeng Yan, Jianpiao Cai, Man Lung Yeung, Jasper Fuk-Woo Chan, Kwok-Yung Yuen

**Affiliations:** 1 State Key Laboratory of Emerging Infectious Diseases, Li Ka Shing Faculty of Medicine, The University of Hong Kong, Pokfulam, Hong Kong China; 2 Department of Microbiology, School of Clinical Medicine, Li Ka Shing Faculty of Medicine, The University of Hong Kong, Pokfulam, Hong Kong China; 3 Centre for Virology, Vaccinology and Therapeutics, Hong Kong Science and Technology Park, Hong Kong, China; 4 Carol Yu Centre for Infection, Li Ka Shing Faculty of Medicine, The University of Hong Kong, Pokfulam, Hong Kong China; 5 Guangzhou Laboratory, Guangdong, China

**Keywords:** Cellular imaging, Molecular biology

## Abstract

The emergence of highly transmissible SARS-CoV-2 variants has led to the waves of the resurgence of COVID-19 cases. Effective antivirals against variants are required. Here we demonstrate that a human-derived peptide 4H30 has broad antiviral activity against the ancestral virus and four Variants of Concern (VOCs) in vitro. Mechanistically, 4H30 can inhibit three distinct steps of the SARS-CoV-2 life cycle. Specifically, 4H30 blocks viral entry by clustering SARS-CoV-2 virions; prevents membrane fusion by inhibiting endosomal acidification; and inhibits the release of virions by cross-linking SARS-CoV-2 with cellular glycosaminoglycans. In vivo studies show that 4H30 significantly reduces the lung viral titers in hamsters, with a more potent reduction for the Omicron variant than the Delta variant. This is likely because the entry of the Omicron variant mainly relies on the endocytic pathway which is targeted by 4H30. Moreover, 4H30 reduces syncytia formation in infected hamster lungs. These findings provide a proof of concept that a single antiviral can inhibit viral entry, fusion, and release.

## Introduction

The SARS-CoV-2 pandemic has lasted for more than 2 years at the time of writing^1^. However, there are few widely available drugs, which could effectively protect humans from SARS-CoV-2 infection. Spike mutations in the Variants of Concern (VOCs) are challenging the efficiency of the first and next generations of vaccines. Patients with severe COVID-19 pneumonia have diffuse alveolar damage in their lung tissues with syncytia formation^2,3^, which was likely attributed to viral spike-ACE2-mediated cell fusion. The syncytia might be related to the inflammatory lung damages in severe COVID-19 patients^4^ and anti-syncytia drugs combined with other antivirals might yield better clinical outcomes^5^. Thus, broad-spectrum antivirals inhibiting viral replication and fusion are still needed for combating SARS-CoV-2 infection.

Antivirals and neutralizing antibodies, which block viral entry into susceptible host cells, can abort the first step of the viral replication cycle. Studies have tried to identify viral entry or fusion inhibitors as the key antiviral strategies^6–9^, including our previous antiviral peptides blocking viral entry by interfering with virus-host endocytosis and cross-linking viral particles^10–13^. SARS-CoV-2 is known to enter cells by binding to Angiotensin-Converting Enzyme 2 (ACE2), heparan sulfate (HS), and other co-entry factors, which allow virus entry through the TMPRSS2-mediated cell surface membrane fusion pathway or the endocytic pathway through the endosomal membrane^14–16^. Though many studies have shown that antibodies, protease inhibitors, and polymerase inhibitors could block SARS-CoV-2 entry or viral RNA synthesis^17–19^, there are limited studies reporting on antiviral inhibition on SARS-CoV-2 release. Antivirals with multiple antiviral functions blocking viral entry, fusion, and release might have potent and broad-spectrum antiviral efficacy in vivo.

In this study, we demonstrated that a peptide 4H30 that was designed from human beta-defensin 2 (HBD2) could significantly inhibit SARS-CoV-2 variants in vitro and in vivo. Mechanistic studies showed that 4H30 could bind to the spike protein of SARS-CoV-2 with cross-linking of viral particles to form big viral clusters. This in turn blocked viral entry in both VeroE6 and Calu-3 cells, which indicated that the cross-linking mechanism of 4H30 effectively blocked both entry pathways of SARS-CoV-2 (namely the endocytic pathway and TMPRSS2-mediated entry pathway). Moreover, 4H30 could inhibit endosomal acidification which therefore blocked spike-ACE2-mediated fusion in endocytosis. Thus, escaped viral particles, which were not clustered and entered into the endosomes, would be stopped at this step of the viral replication cycle. Furthermore, the inhibition of 4H30 on spike-ACE2-mediated fusion could reduce the syncytia formation which may reduce the pathology in vivo. Most interestingly, 4H30 could bind both viral spike and glycosaminoglycans (GAGs) and thus cross-link SARS-CoV-2 with GAGs (heparan sulfate and chondroitin sulfate), which significantly restricted viral release as the last strategy to terminate the viral replication cycle. In vivo studies showed that 4H30 could significantly inhibit SARS-CoV-2 variants when administrated before or after the SARS-CoV-2 challenge in our hamster models. Especially, 4H30 could more effectively inhibit Omicron variant replication in hamsters because 4H30 could inhibit endosomal acidification and the Omicron variant was more dependent on an endocytic pathway for viral infection when compared with the Delta variant. Collectively, we demonstrated a human-derived peptide with triple antiviral mechanisms which act by blocking viral entry, fusion, and release with significant antiviral activity against SARS-CoV-2 variants in vitro and in vivo.

## Results

### Human defensin peptide 4H30 inhibits SARS-CoV-2 replication

**Fig. 1:**
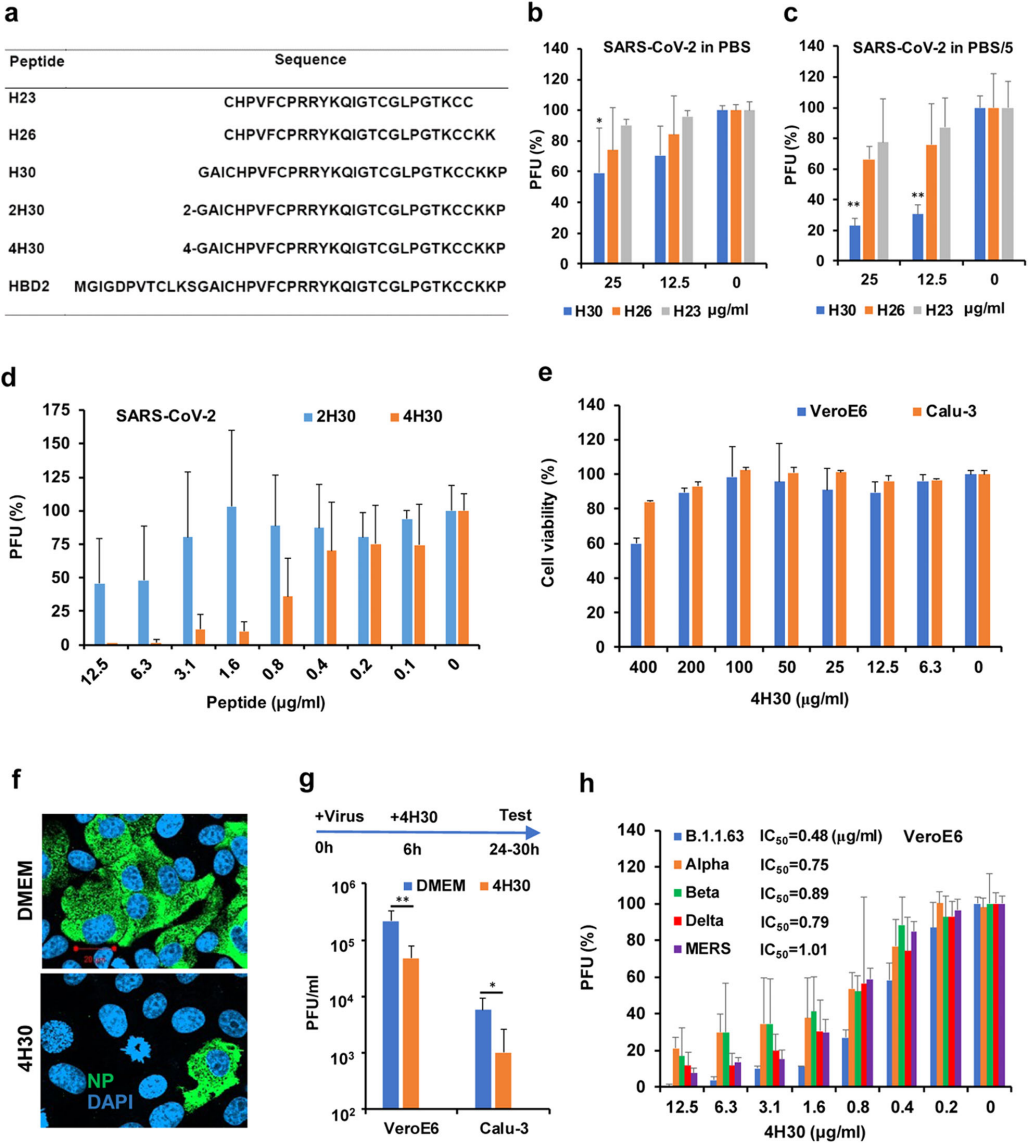
4H30 showed antiviral activity against SARS-CoV-2 before and after virus challenge. **a** Peptide sequences of H23, H26, H30, 2H30, 4H30, and reference HBD2. Peptide 2H30 and 4H30 were 2-branched and 4-branched H30 cross-linked by lysine at the C terminal of H30. **b** The antiviral activity of these peptides in the high salt concentration (150 mM, PBS) was determined by plaque reduction assay (*n* = 3). **c** The antiviral activity of these peptides against SARS-CoV-2 in the low salt concentration (30 mM, PBS/5) was determined by plaque reduction assay (*n* = 3). *P* values were generated by comparison with an untreated virus (0). **d** The antiviral activity of 2-branched H30 (2H30) and 4-branched H30 (4H30) against SARS-CoV-2 (HKU001a) in PBS was determined by plaque reduction assay (*n* = 4). **e** The cytotoxicity of 4H30 in VeroE6 and Calu-3 cells was measured by MTT assay (*n* = 3). **f** The antiviral activity of 4H30 was determined by anti-nucleocapsid (NP) immunofluorescent staining. SARS-CoV-2 with or without 4H30 treatment was added to cells for infection. Representative images were taken at 18 h post-infection (hpi) in VeroE6 cells. Scale bar, 20 μm. **g** The post-infection antiviral activity of 4H30 against SARS-CoV-2 (B.1.1.63, D614G) in VeroE6 and Calu-3 cells (*n* = 6). At 6 hpi, 4H30 (50 μg/mL) was added to infected cells and viral titers in cell supernatants were measured at 24 hpi (for VeroE6 cells) or 30 hpi (for Calu-3 cells). **h** The broad-spectrum antiviral activities of 4H30 against SARS-CoV-2 variants and MERS-CoV (MERS) in VeroE6 cells were determined by plaque reduction assay (*n* = 4). *P < 0.05 and **P < 0.01 when compared with DMEM. *P* values were calculated by the two-tailed Student’s *t*-test. Data were presented as means ± SD of indicated biological samples with more than two independent experiments.

HBD2 was demonstrated to have broad antimicrobial activities^20^. However, defensin peptides generally showed decreased antimicrobial activity in the high salt condition when compared with their activity in the low salt condition^10,21^. Decreased antimicrobial activity of defensin peptides in the high salt condition poses a barrier to developing them as antimicrobials in vivo despite their broad-spectrum antimicrobial activities in vitro. To find a human-sourced antiviral peptide with effective antiviral activity in the high salt condition, we first designed short H23, H26, and H30 peptides based on HBD2 and we demonstrated that H30 could inhibit SARS-CoV-2 in phosphate-buffered saline (PBS) but more effective in the low salt condition (Fig. 1a–c). The weak antimicrobial activity of H30 in PBS was consistent with the decreased antimicrobial activity of HBD2 and other defensins in the high salt condition^21–23^. Next, we synthesized two-branched H30 (2H30) and four-branched H30 (4H30). The antiviral assays indicated that 4H30 could significantly inhibit SARS-CoV-2 infection (the 50% inhibitory concentration: IC_50_ = 0.59 μg/mL or 44 nM) which was more potent than H30 (IC_50_ > 25 μg/mL) and 2H30 (Fig. 1d) in PBS. 4H30 significantly inhibited SARS-CoV-2 in the low salt condition (Supplementary Fig. S1a). The cytotoxicity assay indicated that the 50% toxicity concentration (TC_50_) of 4H30 in VeroE6 and Calu-3 cells was higher than 400 μg/mL (Fig. 1e). To confirm the antiviral activity of 4H30, the anti-nucleocapsid staining result demonstrated that viral replication was significantly inhibited and restricted in very few cells when compared with an untreated virus (Fig. 1f and Supplementary Fig. S1b). Furthermore, we demonstrated that 4H30 could significantly inhibit SARS-CoV-2 replication in VeroE6 and Calu-3 cells when 4H30 was added to cells at 6 h post-infection (hpi) (Fig. 1g). To investigate the efficacy of 4H30 against SARS-CoV-2 variants, we showed that 4H30 could significantly inhibit the replication of four SARS-CoV-2 variants and MERS-CoV in VeroE6 cells by plaque reduction assay (Fig. 1h). These results indicated that branched 4H30 could potently inhibit SARS-CoV-2 replication when treated with 4H30 before or after virus challenge in the high salt concentration.

### 4H30 blocks SARS-CoV-2 entry

**Fig. 2:**
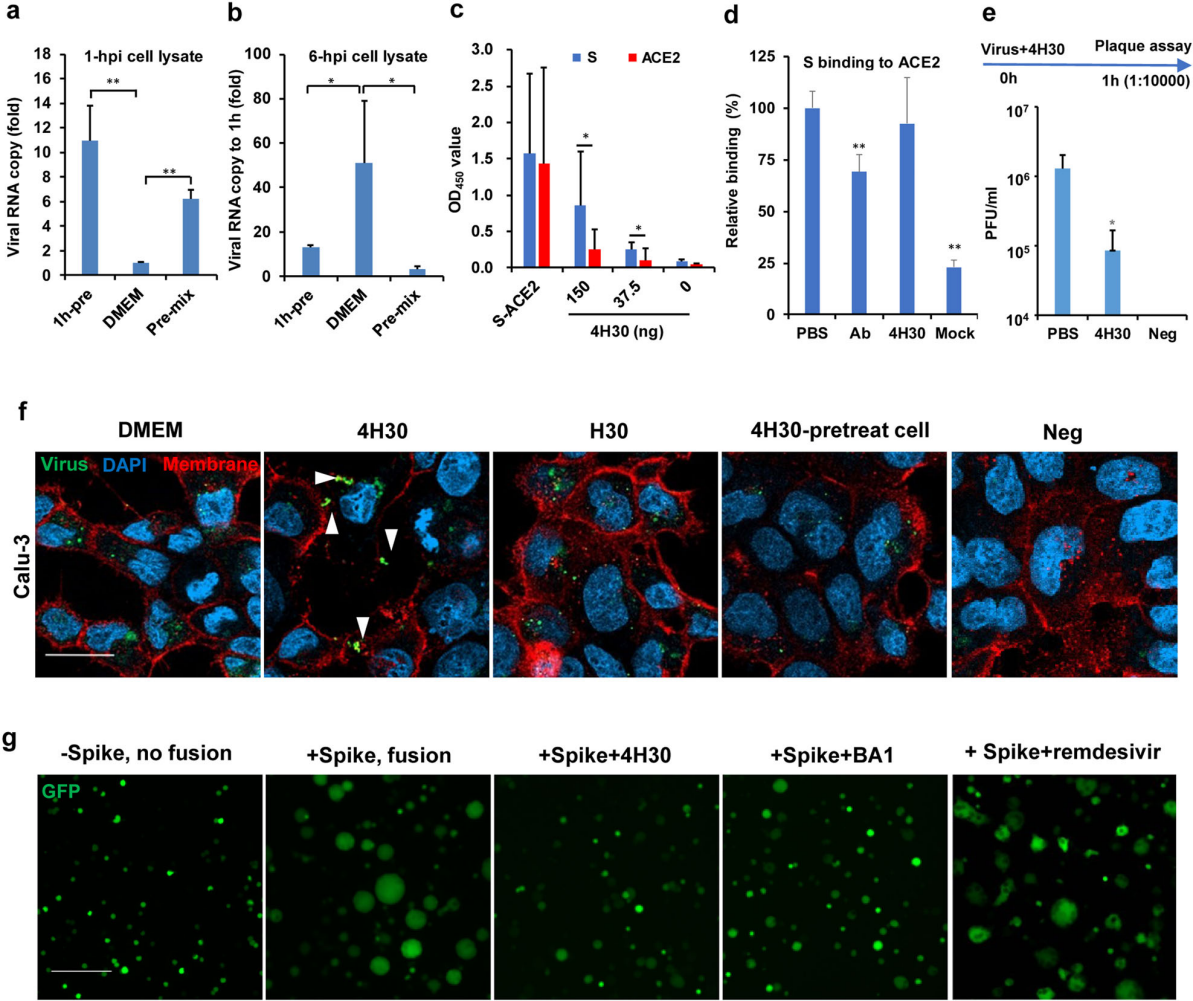
4H30 could cluster virus together to block viral entry and fusion. **a** 4H30 increased virus attachment to VeroE6 cells. When cells were pretreated by 4H30 (25 μg/mL) 1 h before viral infection (1h-Pre) or when the virus was pretreated by 4H30 before viral infection (Pre-mix), the viral RNA copies in infected cell lysate were measured at 1 hpi. RNA copy (fold) was normalized to DMEM-treated virus (*n* = 4). **b** 4H30 could inhibit viral replication at 6 hpi when the virus was pretreated by 4H30 (*n* = 4). Viral loads in cell lysate were measured by RT-qPCR and viral RNA copy was normalized to that of 1 hpi, correspondingly. *P* values were generated by comparison with DMEM. **c** 4H30 (150, 37.5, or 0 ng) could more effectively bind to spike when compared with ACE2 (*n* = 8). Spike or ACE2 (100 ng) was added to coated 4H30 on the ELISA plate. Spike and ACE2 (S-ACE2, 100 ng) coated on an ELISA plate were the positive controls. *P < 0.05 when compared with ACE2. **d** 4H30 did not reduce ACE2 binding to spike (*n* = 6). Spike bound to ACE2 (150 ng) coated on ELISA plate. Ab, neutralizing antibody of the spike. **P < 0.01 when compared with PBS. **e** 4H30 (50 μg/mL) could significantly inhibit SARS-CoV-2 when 4H30-treated virus (10^6^ PFU/mL) was diluted by 10,000 folds for plaque reduction assay (*n* = 4). Cells without infection was the negative (Neg) control. *P < 0.05 when compared with PBS. **f** 4H30 could cross-link SARS-CoV-2 to form big viral clusters and block virus entry through the Calu-3 cell membrane. SARS-CoV-2 (green) was treated by DMEM, 4H30 or H30 before infecting cells, or cells were treated by 4H30 before viral challenge (4H30-pretreat cell). Cells without infection was the negative (Neg) control. Representative images were taken at 1 hpi in Calu-3 cells. White triangles indicated the clustered viral particles. Scale bar, 20 μm. **g** 4H30 could inhibit spike-ACE2-mediated cell fusion in 293T cells. 293T-ACE2 cells were cocultured with 293T with a spike (+Spike) or without spike (–Spike) under the treatment of 4H30 (40 μM), bafilomycin A1 (50 nM), or remdesivir (80 μM). Representative images were taken at 6 h post coculture. Fused cells (+Spike, > 50 μm) were larger than unfused cells (–Spike, ~10 μm), the 4H30-treated cells (+Spike+4H30) or BA1-treated cells. Scale bar, 200 μm. *P* values were calculated by the two-tailed Student’s *t*-test. Data were presented as means ± SD of indicated biological samples with more than two independent experiments.

To investigate the antiviral mechanism of 4H30, we showed that viral RNA copies in VeroE6 cells were significantly increased at 1 hpi after washing the infected cells when cells were pretreated by 4H30 or when the virus was pretreated by 4H30 (Fig. 2a). At 6 hpi, virus replication in cell lysate was significantly inhibited when the virus was pretreated by 4H30 as demonstrated by less than threefold RNA increase when compared with viral load at 1 hpi (Fig. 2b), which was consistent with plaque reduction assay when the virus was pretreated by 4H30 (Fig. 1d, h). However, Dulbecco’s Modified Eagle Medium (DMEM)-treated virus showed a 50-fold viral load increase at 6 hpi when compared with viral load at 1 hpi. Compared with the viral load at 1 hpi, there was a 13-fold viral load increase at 6 hpi when cells were pretreated by 4H30 before viral challenge (1h-pre), which indicated that the increased viral infection at 1 hpi (1h-pre) did not increase the viral replication at 6 hpi (1h-pre) when compared with DMEM-treated virus (Fig. 2b). Next, we showed that 4H30 could increase SARS-CoV-2 attachment to VeroE6 cells at 4 °C (Supplementary Fig. S2), which was consistent with the increased viral load at 1 hpi (Fig. 2a). Furthermore, 4H30 could more effectively bind to the spike when compared with ACE2 (Fig. 2c and Supplementary Fig. S3), while 4H30 did not directly affect the binding between the spike and ACE2 (Fig. 2d). These results indicated that 4H30 did not directly cross-link spike with ACE2 to increase viral attachment because 4H30 could bind to spike but not effectively bind to ACE2. Moreover, when 1 × 10^6^ plaque-forming unit/mL (PFU/mL) of SARS-CoV-2 was pretreated by 50 μg/mL of 4H30 and then the 4H30-treated virus was 10,000-fold diluted for plaque assay, 4H30 (at 0.005 μg/mL < IC_50_ = 0.59 μg/mL) could significantly inhibit SARS-CoV-2 infection (Fig. 2e), which indicated that 4H30 could inhibit viral infection by binding to virus before viral entry. Collectively, these results indicated that 4H30 could increase viral attachment to cells but still effectively inhibit viral replication in the following steps of viral entry.

Next, we showed that 4H30 could cross-link SARS-CoV-2 to form big viral clusters on Calu-3 (Fig. 2f) and VeroE6 (Supplementary Fig. S4) cell membrane without viral entry, which indicated that cross-linked viral particles could not infect cells through the two entry pathways of SARS-CoV-2 mediated by surface membrane fusion in Calu-3 cells with TMPRSS2^+^ or by endocytosis in VeroE6 cells. To further confirm that 4H30 could cluster viral particles, we demonstrated that 4H30 could cross-link SARS-CoV-2 to form bigger viral clusters under transmission electron microscopy (TEM) (Supplementary Fig. S5). The intact viral particles under TEM indicated that 4H30 did not disrupt viral particles. Because 4H30 could bind to spike (Fig. 2c), it is conceivable that 4H30 could also cross-link spike protein as shown under TEM (Supplementary Fig. S6). When Calu-3 cells or VeroE6 cells were pretreated by 4H30 before virus challenge (4H30-pretreat cell), 4H30 did not cluster viral particles and did not block viral entry (Fig. 2f; Supplementary Fig. S4), which indicated that 4H30 did not affect host factors such as ACE2 on cell membrane to inhibit viral entry. Collectively, these results indicated that 4H30 could cross-link SARS-CoV-2 through its binding to spike to form big viral particles and therefore block viral entry through both pathways.

### 4H30 inhibits spike-ACE2-mediated fusion

Since our previous studies showed that basic virus-binding peptides could inhibit endosomal acidification and spike-ACE2-mediated cell membrane fusion^10,11^, we hypothesized that basic 4H30 could also inhibit spike-ACE2-mediated fusion. First, we cocultured spike-expressing 293T cells and ACE2-expressing 293T cells and observed fusion mediated by the endocytic pathway^15^. 4H30 could inhibit the fusion in this 293T-Spike+293T-ACE2 system (Fig. 2g), which was similar to the inhibition of bafilomycin A1 on the fusion. The 293T-Spike cells expressing GFP could fuse with 293T-ACE2 cells to allow the entry of GFP into multiple cells fused together to form large cells with high GFP expression. The basic 4H30 and the bafilomycin A1 could inhibit the endosomal acidification in live cells (Supplementary Fig. S7), which was consistent with our previous study of the basic peptide P9R preventing endosomal acidification to block spike mediated fusion^11^, and thus both could block spike-ACE2-mediated fusion in 293 T cells (Fig. 2g). Next, we cocultured spike-expressing 293 T cells with Calu-3 cells, in which the TMRSS2-meditated cell surface fusion was not affected by endocytosis. In this 293T-Spike+Calu-3 system, 4H30 and bafilomycin A1 could not inhibit cell-cell fusion (Supplementary Fig. S8). Taken together, our results suggested that 4H30 prevented spike-ACE2-mediated fusion via the endocytic pathway.

### 4H30 blocks SARS-CoV-2 release

**Fig. 3:**
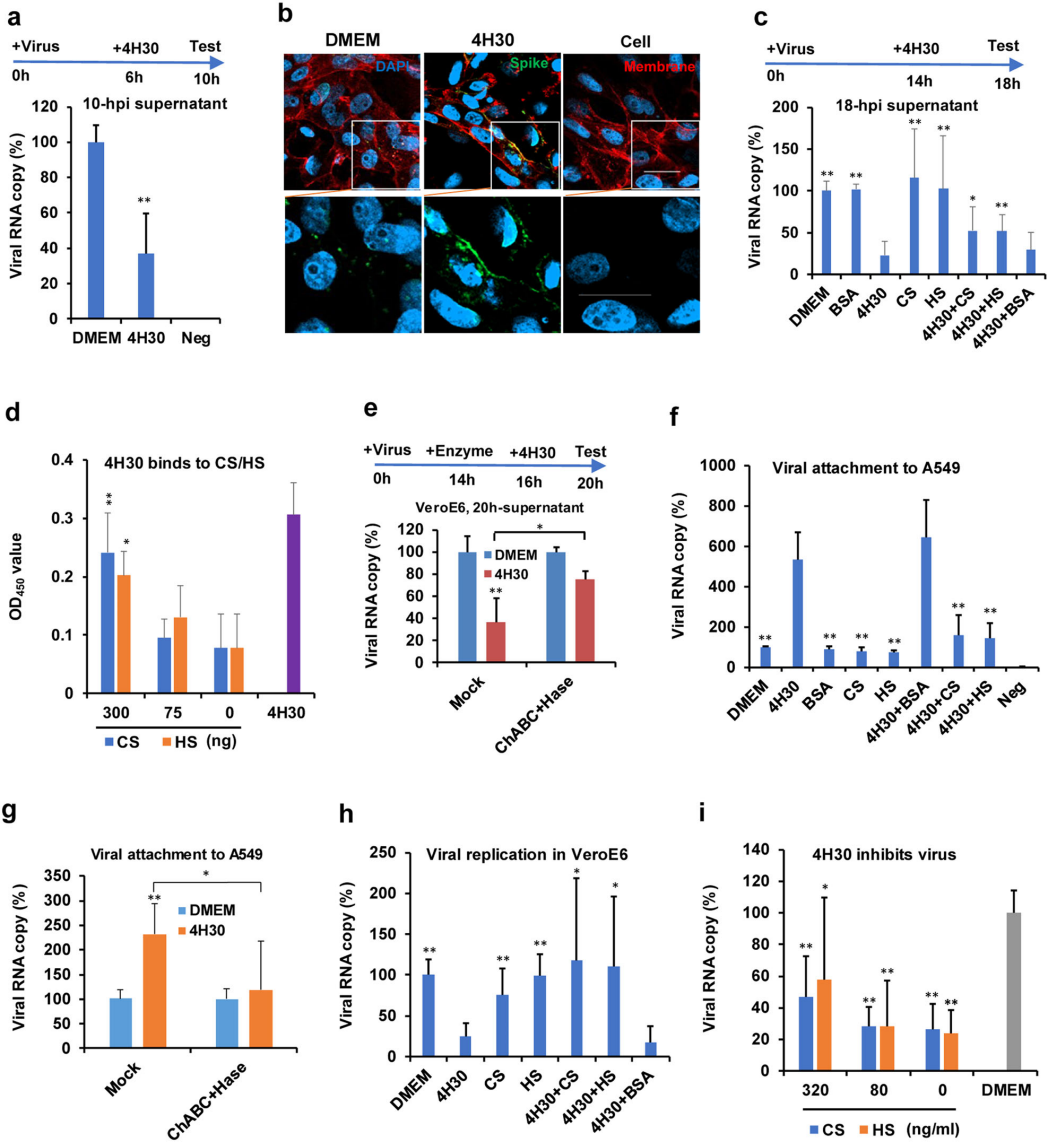
4H30 could inhibit SARS-CoV-2 release. **a** 4H30 could inhibit SARS-CoV-2 release at 10 hpi (*n* = 6). 4H30 was added to infected cells at 6 hpi. The supernatant viral titers were measured at 10 hpi. Viral RNA copy (%) was normalized to DMEM. Cells without infection was the negative (Neg) control. **P < 0.01 when compared with DMEM. **b** 4H30 inhibited SARS-CoV-2 release at 18 hpi as determined by anti-spike staining. Blue, nuclear. Red, membrane staining. Green, anti-spike staining. Scale bar, 20 μm. **c** Chondroitin sulfate (CS) and heparan sulfate (HS) could reduce the inhibition of 4H30 on virus release at 18 hpi (*n* = 6). The virus was treated with the indicated peptides or glycosaminoglycans (CS or HS) at 14 hpi and viral loads in culture supernatants were measured by RT-qPCR at 18 hpi. 4H30 + CS: 4H30 was premixed with CS. 4H30 + HS: 4H30 was premixed with HS. 4H30 + BSA: 4H30 was premixed with BSA for 30 min before adding to the virus. Viral RNA copy (%) was normalized to DMEM. **P < 0.01 and *P < 0.05 when compared with 4H30. **d** 4H30 could bind to CS and HS (*n* = 4). CS or HS (300, 75, 0 ng = BSA) was coated on ELISA plate. 4H30 binding to CS or HS was measured by anti-HBD2. 4H30 coated on an ELISA plate was used as the positive control. **P < 0.01 when compared with “0”. **e** Cleaving CS and HS by chondroitinase ABC (ChABC) and Heparanase (Hase) could reduce the inhibition of 4H30 on viral release (*n* = 4). ChABC and Hase were added to infected VeroE6 cells at 14 hpi for removing CS and HS at 37 °C for 2 h and then 4H30 was added to cells at 16 hpi and viral titers in supernatants were measured by RT-qPCR at 20 hpi. **f** 4H30 increasing SARS-CoV-2 attachment to A549 cells at 4 °C could be reduced by CS or HS treatment (*n* = 5). Virus attachment to A549 cells of SARS-CoV-2 treated by DMEM, 4H30, BSA, CS, HS, 4H30 + BSA, 4H30 + CS, and 4H30 + HS was measured by RT-qPCR. Cells without infection was the negative (Neg) control. Viral RNA copy (%) was normalized to DMEM. **P < 0.01 when compared with 4H30. **g** The attachment of SARS-CoV-2 to 4H30-treated A549 cells could be reduced by ChABC and Hase (*n* = 8). A549 cells were pretreated by buffer (Mock) or ChABC+Hase and then further treated by DMEM or 4H30. SARS-CoV-2 attachment to the treated cells was measured by RT-qPCR. Viral RNA copy (%) was normalized to DMEM. **P < 0.01 when compared with DMEM. **h** High concentration of CS and HS (1250 ng/mL) could reduce the antiviral activity of 4H30 in VeroE6 (*n* = 6). **P < 0.01 when compared with 4H30. **i** 4H30 could significantly inhibit viral replication in the presence of a low concentration of CS or HS (320, 80 or 0 ng/mL). Virus with the indicated treatment grew in VeroE6 cells and the supernatant virus was measured by RT-qPCR at 24 hpi (*n* = 6). **P < 0.01 and *P < 0.05 when compared with DMEM. *P* values were calculated by the two-tailed Student’s *t*-test. Data were presented as means ± SD of indicated biological samples with more than two independent experiments.

Notably, we demonstrated that 4H30 could significantly inhibit viral release as indicated by the significantly reduced viral load of SARS-CoV-2 in cell culture supernatants at 10 hpi when 4H30 was added to infected cells at 6 hpi (Fig. 3a) and the inhibitory effect of 4H30 was not on the intracellular replicative process during this short 4-h treatment (Supplementary Fig. S9). To confirm that 4H30 inhibited viral release, we assessed the clumping of spike protein on the cell surface by confocal microscopy. When compared with DMEM-treated infected cells, the spike protein could be more clearly seen on the cell surface when 4H30 was added to infected cells at 14 hpi and then the infected cells were fixed at 18 hpi for anti-spike staining (Fig. 3b). The significant decrease of viral load in culture supernatants at 18 hpi further confirmed that 4H30 significantly inhibited SARS-CoV-2 release when compared with BSA-treated or DMEM control (Fig. 3c). Previous studies showed that HBD2 could bind to cellular GAGs^24^. Hence, we hypothesized that 4H30 could inhibit the release of the mature virion by cross-linking the virion with GAGs. We showed that 4H30 could effectively bind to GAGs including heparan sulfate (HS) and chondroitin sulfate (CS) when compared with BSA (Fig. 3d). In addition, we showed that the antiviral activity of 4H30 on viral release was significantly reduced when 4H30 was premixed with HS or CS (Fig. 3c), but BSA did not significantly affect the inhibitory activity of 4H30 on SARS-CoV-2 release (Fig. 3c). Moreover, when VeroE6 cells were treated with chondroitinase ABC (ChABC) and heparinase (Hase) to remove cell surface CS and HS, the inhibition of 4H30 on viral release was significantly reduced (Fig. 3e). The attachment of SARS-CoV-2 to cells treated by these enzymes was also significantly reduced (Supplementary Fig. S10), which indicated that cleaving the attachment factor HS^14^ could reduce the attachment of SARS-CoV-2 to cells. These results indicated that 4H30 could cross-link SARS-CoV-2 with cell surface HS and CS to inhibit viral release.

### 4H30 affects SARS-CoV-2 attachment

Because 4H30 could cross-link virus with GAGs to affect viral release, we asked if 4H30 could also affect viral attachment by cross-linking virus with cell surface GAGs. We demonstrated that the attachment of SARS-CoV-2 to cells treated by 4H30 was significantly increased in A549 (ACE2^–^)^25^ and Calu-3 (ACE2^+^) cells when compared with BSA-treated or DMEM-treated cells (Fig. 3f and Supplementary Fig. S11). The increased virus attachment did not increase SARS-CoV-2 replication in Calu-3 cells (Supplementary Fig. S12), which was consistent with the viral replication results in VeroE6 cells when cells were pretreated by 4H30 (Fig. 2b). We further demonstrated that the attachment was significantly reduced when 4H30 was pretreated by 5 μg/mL of CS or HS (Fig. 3f) and when A549 cells were treated with ChABC and Hase which could remove cell surface CS and HS (Fig. 3g). Taken together, these results further confirmed that 4H30 could cross-link virus with cell surface GAGs to affect viral attachment and release even in the absence of ACE2 in A549 cells.

Moreover, we demonstrated that the antiviral activity of 4H30 treated with 1250 ng/mL of CS or HS (Fig. 3h) was significantly reduced when compared with untreated 4H30 or BSA-treated 4H30. However, when CS or HS was in the physiological concentration (50–100 ng/mL)^26^, CS/HS-treated 4H30 could still significantly inhibit viral replication (Fig. 3i), which was consistent with the antiviral activity of 4H30 when it was added to cells after viral infection (Fig. 1g). Together with its inhibition of viral entry and spike-ACE2-mediated fusion, these results showed that 4H30 could cross-link viral particles and bind to viral spike and cellular GAGs to achieve multiple antiviral activities through the triple-functionality effects of blocking viral entry, fusion, and release.

### Toxicity of 4H30 in vivo

**Fig. 4:**
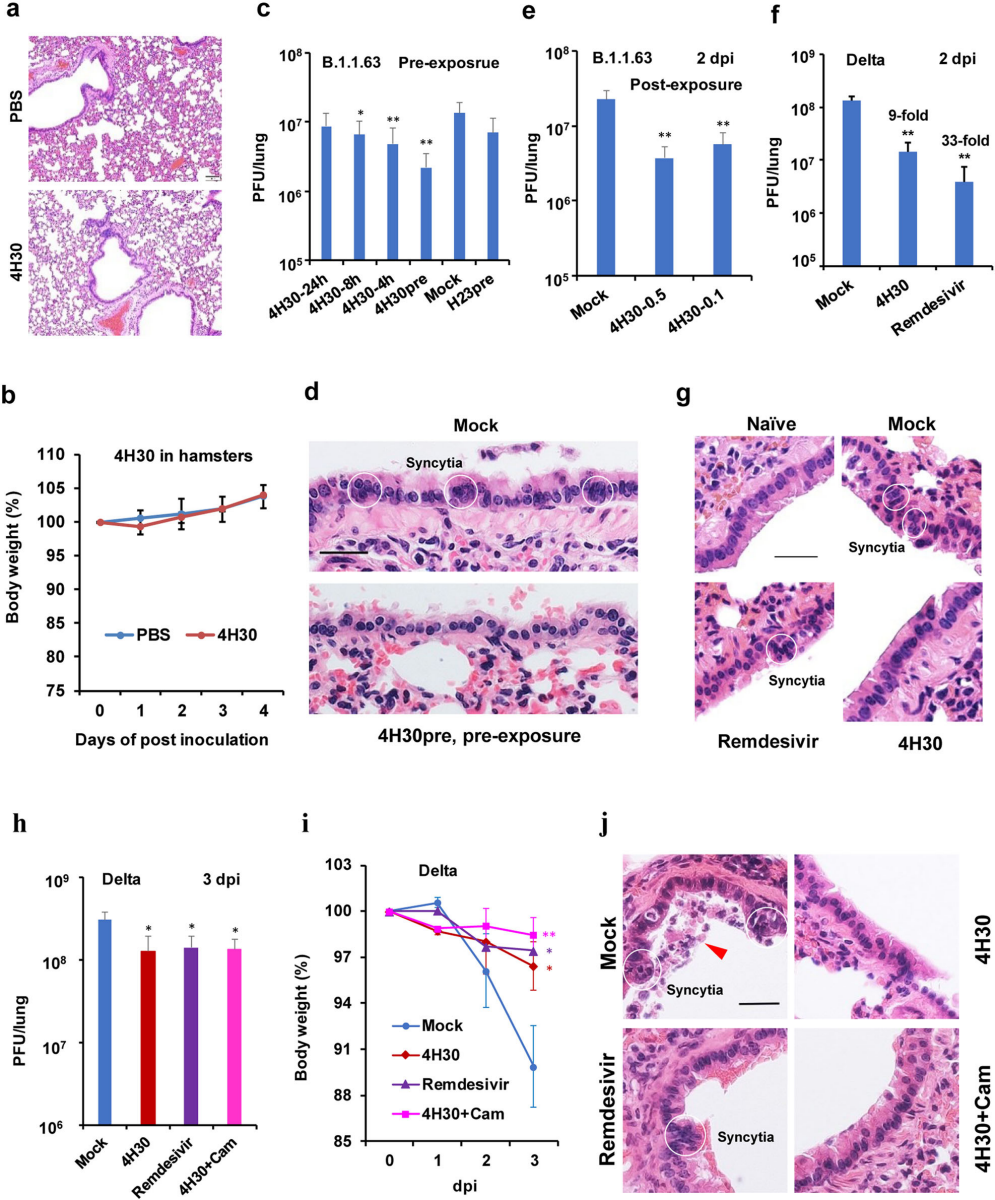
4H30 could broadly inhibit SARS-CoV-2 variants in hamsters. **a**, **b** Histopathology of hamster lungs (**a**) and body weight of hamsters (**b**) treated by 4H30 or PBS. Three intranasal doses of 4H30 (0.5 mg/kg) were administered within two days. The lung tissues were harvested on day 4 post-inoculation for H&E staining. Scale bar, 100 μm. **c** 4H30 showed pre-exposure activity to significantly inhibit SARS-CoV-2 (B.1.1.63) replication. Intranasal 4H30 (0.5 mg/kg) was administered to hamsters at 24 h (*n* = 4), 8 h (*n* = 4), 4 h (*n* = 6), or before virus challenge (4H30pre, *n* = 4). Intranasal H23 was administered to hamsters before the virus challenge (H23pre). Virus titers in the lungs were measured at 2 dpi. **P < 0.01 when compared with PBS-treated hamsters (Mock). **d** 4H30pre could reduce syncytia formation in the bronchial epithelium of hamsters when compared with mock-treated hamsters. White circles indicated the syncytial cells with 4 or more nuclei. Scale bar, 25 μm. **e** 4H30 showed post-exposure activity to inhibit SARS-CoV-2 (B.1.1.63) replication (*n* = 4). 4H30 (0.5 or 0.1 mg/kg) or PBS (Mock) was intranasally inoculated into hamsters at 8 hpi. Virus titers were measured at 2 dpi. **f** 4H30 and remdesivir could significantly inhibit SARS-CoV-2 (B.1.617.2) replication in hamster lungs (*n* = 4). Intranasal 4H30 or remdesivir (0.5 mg/kg) were administered to hamsters at 8 hpi and two more doses were given to hamsters in the following day. Virus titers were measured at 2 dpi. **P < 0.01 when compared with Mock-treated hamsters. **g** 4H30 could reduce syncytia formation in the bronchial epithelium of hamsters challenged by the SARS-CoV-2 Delta variant (B.1.617.2). Representative images were taken at 2 dpi. Scale bar, 25 μm. **h**–**j** Anti-Delta activity at 3 dpi. Infected hamsters were intranasally inoculated with PBS (Mock, *n* = 4), 4H30 (*n* = 3), remdesivir (*n* = 3) or 4H30 + Cam (*n* = 3, camostat = 1 mg/kg) at 8 hpi. Viral titers (**h**), body weight (**i**), and histopathology (**j**) were tested at 3 dpi. *P < 0.05 and **P < 0.01 when compared with Mock. Scale bar, 25 μm. The red triangle indicated the desquamation of the epithelial cells. White circles indicated the syncytial cells with six or more nuclei. Data were presented as means ± SD of indicated independent biological samples.

To test for potential toxicity of 4H30 in vivo, we monitored the pathological change and body weight change after intranasal administration of 4H30 into hamsters or mice. Inhaled 4H30 did not cause significant pathological and body weight changes in hamster lungs at 4 days post-inoculation (Fig. 4a, b and Supplementary Fig. S13). To determine if 4H30 could cause any lung damage over a longer period, we monitored the lung histopathology and body weight changes for 40 days. There was no significant histopathological and consistent body weight change when compared with the PBS group (Supplementary Fig. S14).

### 4H30 against SARS-CoV-2 variants in hamsters

First, we tested the in vivo pre-exposure activity of 4H30 against SARS-CoV-2 (B.1.1.63, a D614G virus). The lung viral load reduction (6.3-fold) was greatest when only one dose (0.5 mg/kg) of 4H30 was administered to hamster lungs just before infection (Fig. 4c, 4H30pre), but the control peptide H23pre did not show significantly antiviral activity. The lung viral load reduction (2.1-fold) was observed even when 4H30 was administered at 8 h prior to infection (4H30-8h). In addition, syncytia were found in the bronchial tree epithelial cells in the mock-treated hamster lungs, but this histopathological change of syncytia was reduced in hamsters treated by 4H30pre (Fig. 4d). This result was corroborated with our findings of in vitro fusion assays in Fig. 2g, which showed that 4H30 could inhibit spike-ACE2-mediated cell-cell fusion.

To investigate the post-exposure antiviral activity of 4H30 in vivo, we challenged hamsters with SARS-CoV-2 (B.1.1.63) and then treated hamsters by intranasal inhalation of 4H30 (0.5 or 0.1 mg/kg) or PBS. Two more doses were given to hamsters on the following day. Because SARS-CoV-2 replicated quickly in hamster lungs and reached the peak titers at 2 dpi^27,28^, we started the first dose at 8 hpi and viral loads in lungs were measured at 2 dpi (Fig. 4e). 4H30 (0.5 and 0.1 mg/kg) could significantly inhibit SARS-CoV-2 replication when compared with mock-treated hamsters. We further confirmed that 4H30 (0.5 mg/kg = 0.04 μmol/kg) could significantly inhibit SARS-CoV-2 (B.1.617.2, Delta) replication in hamster lungs (Fig. 4g) and remdesivir^29^ with the dosage (0.5 mg/kg = 0.8 μmol/kg) could also significantly inhibit SARS-CoV-2 replication. Interestingly, 4H30 could reduce the syncytia formation in hamster lungs (Fig. 4h) when compared with mock-treated hamsters. However, syncytia could be easily observed in the bronchi epithelial cells of hamster lungs treated by remdesivir even though remdesivir could inhibit 33-fold viral replication in hamster lungs when compared with 9-fold inhibition by 4H30, which was consistent with the antiviral mechanism of remdesivir interfering with viral RNA synthesis without inhibition on spike-ACE2-mediated cell fusion (Fig. 2g).

To further investigate and confirm the protection of fusion inhibition activity on infected hamsters, we treated hamsters with 4H30, remdesivir, or the combination of 4H30 with camostat, which inhibited TMPRSS2 but not significantly inhibited SARS-CoV-2 in hamsters alone^10^. Because SARS-CoV-2 (Delta) already reached the peak titers at 2 dpi, as we expected, all treatments could only inhibit (2–3-fold) viral replication at 3 dpi (Fig. 4h), which was less than the virus-inhibition fold at 2 dpi (9–33-fold). Notably, all treatments could significantly reduce the body weight loss at 3 dpi (Fig. 4i), and especially hamsters treated by 4H30 plus camostat showed the least body weight loss. Histopathology assay showed that syncytia (a large cell with abundant cytoplasm containing multiple nuclei), desquamation of the epithelial cells (Fig. 4j), and inflammation changes (Supplementary Fig. S15) were observed in mock-treated lungs, while big syncytia (> 6 nuclei syncytia) was easily observed in remdesivir-treated hamster lungs (Fig. 4j). 4H30, remdesivir or 4H30 plus camostat could reduce lung inflammation (Supplementary Fig. S15). The least amount of body weight loss and reduced inflammation was observed in hamsters treated with 4H30 plus camostat, indicating that blocking both fusion pathways (endocytosis and TMPRSS2) by 4H30 and camostat could reduce the pathogenicity of Delta variant in lungs.

### 4H30 effectively inhibits the Omicron variant in hamsters

**Fig. 5:**
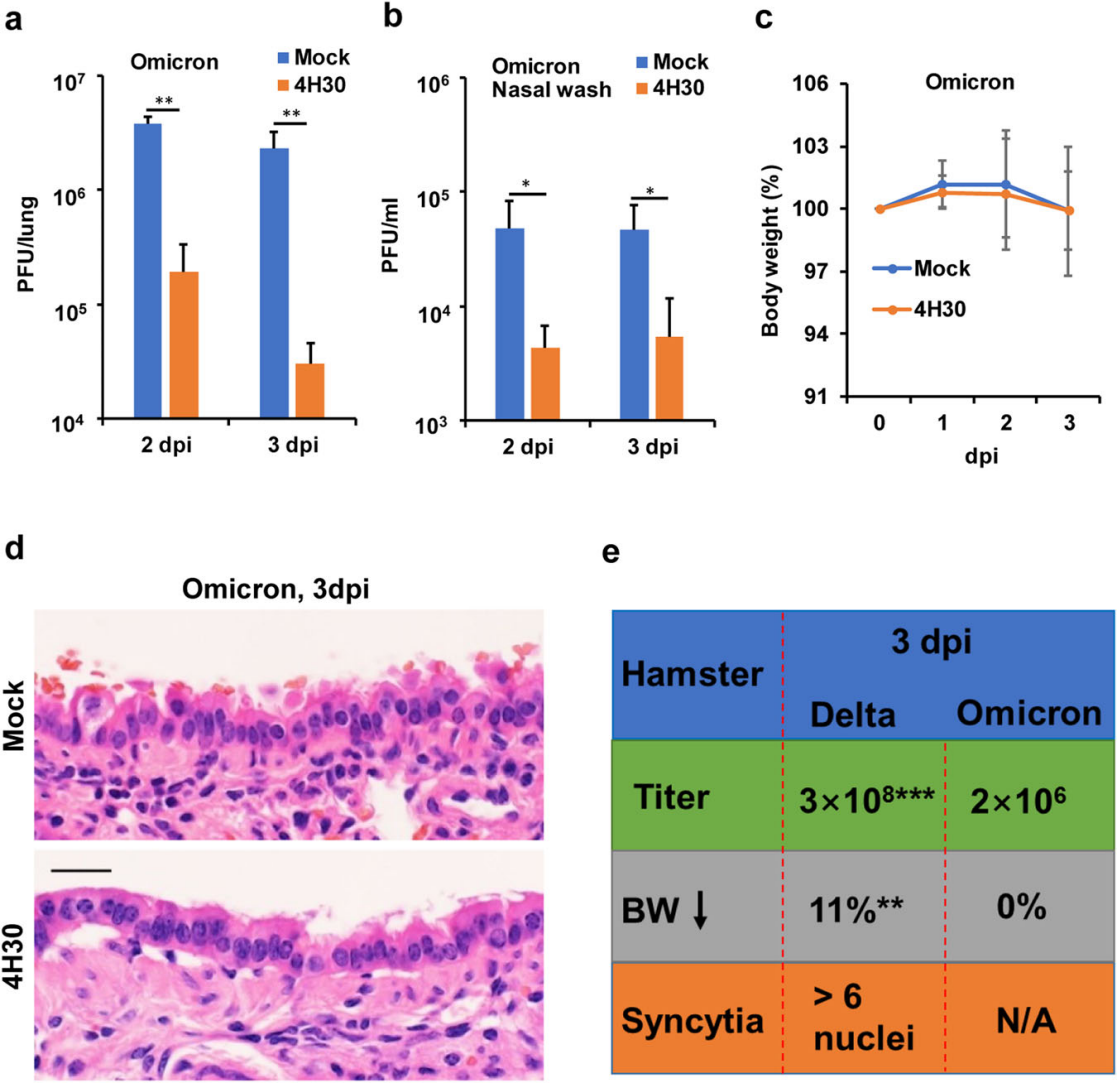
4H30 significantly inhibited Omicron variant (B.1.1.529) replication in hamsters. **a** 4H30 inhibited the replication of Omicron variant in hamster lungs at 2 dpi (*n* = 3) and 3 dpi (*n* = 4). **b** 4H30 inhibited the replication of the Omicron variant in the nasal cavity. Viral titers in the nasal wash were determined by plaque assay at 2 dpi and 3 dpi (*n* = 4). **P < 0.01 when compared with Mock. **c** The body weight of Omicron-infected hamsters treated with 4H30 or PBS (Mock). **d** The histopathology of hamster lungs infected with Omicron variant at 3 dpi. Scale bar, 20 μm. **e** Comparison of viral titer (PFU/lung), body weight loss (BW), and syncytia formation in hamsters infected with Delta or Omicron variant without treatment at 3 dpi. N/A, not applicable. **P < 0.01 and ***P < 0.001 when compared with Omicron.

We recently identified the most recent Omicron variant which did not effectively use TMPRSS2 to support viral replication in Calu-3 cells and was less sensitive to camostat (TMPRSS2 inhibitor) in VeroE6/TMPRSS2 cells^30^. Thus, we hypothesized that 4H30, which inhibited endocytosis but did not inhibit the TMPRSS2-mediated fusion (Supplementary Fig. S8), might more effectively inhibit the Omicron variant in vivo. We demonstrated that 4H30 could significantly inhibit more than 20-fold Omicron replication in hamster lungs at 2 dpi and 3 dpi (Fig. 5a), while 4H30 showed less than ninefold inhibition on Delta variant in hamster lungs at 2 dpi (Fig. 4f) and 3 dpi (Fig. 4h). In addition, 4H30 could significantly reduce Omicron variant titers in the nasal wash at 2 dpi and 3 dpi (Fig. 5b), which indicated the significant antiviral activity of 4H30 in the upper respiratory tract.

Next, we tried to determine the protection of 4H30 on hamsters infected with the Omicron variant. However, even untreated hamsters infected with the Omicron variant did not exhibit significant weight loss at 3 dpi (Fig. 5c) or lung damage (Fig. 5d). We were not able to assess the difference of body weight loss and syncytia formation between 4H30-treated or untreated hamsters infected with the Omicron variant, while there were significant differences when compared with the Delta variant (Fig. 5e).

Taken together, we demonstrated that 4H30 could broadly inhibit viral replication of SARS-CoV-2 variants with reduced inflammation and syncytia formation in hamster lungs and that 4H30 could more effectively inhibit Omicron replication in hamsters when compared with the Delta variant. The reduced syncytia may decrease the damage of SARS-CoV-2 infection in vivo because patients who died from severe COVID-19 had syncytial changes in the lungs at autopsy^31^. The different preferences of infectious pathways between Omicron and Delta variants could affect the pathogenesis and the efficiency of antivirals, which provided the information for evaluating clinical manifestations and treatment.

## Discussion

**Fig. 6:**
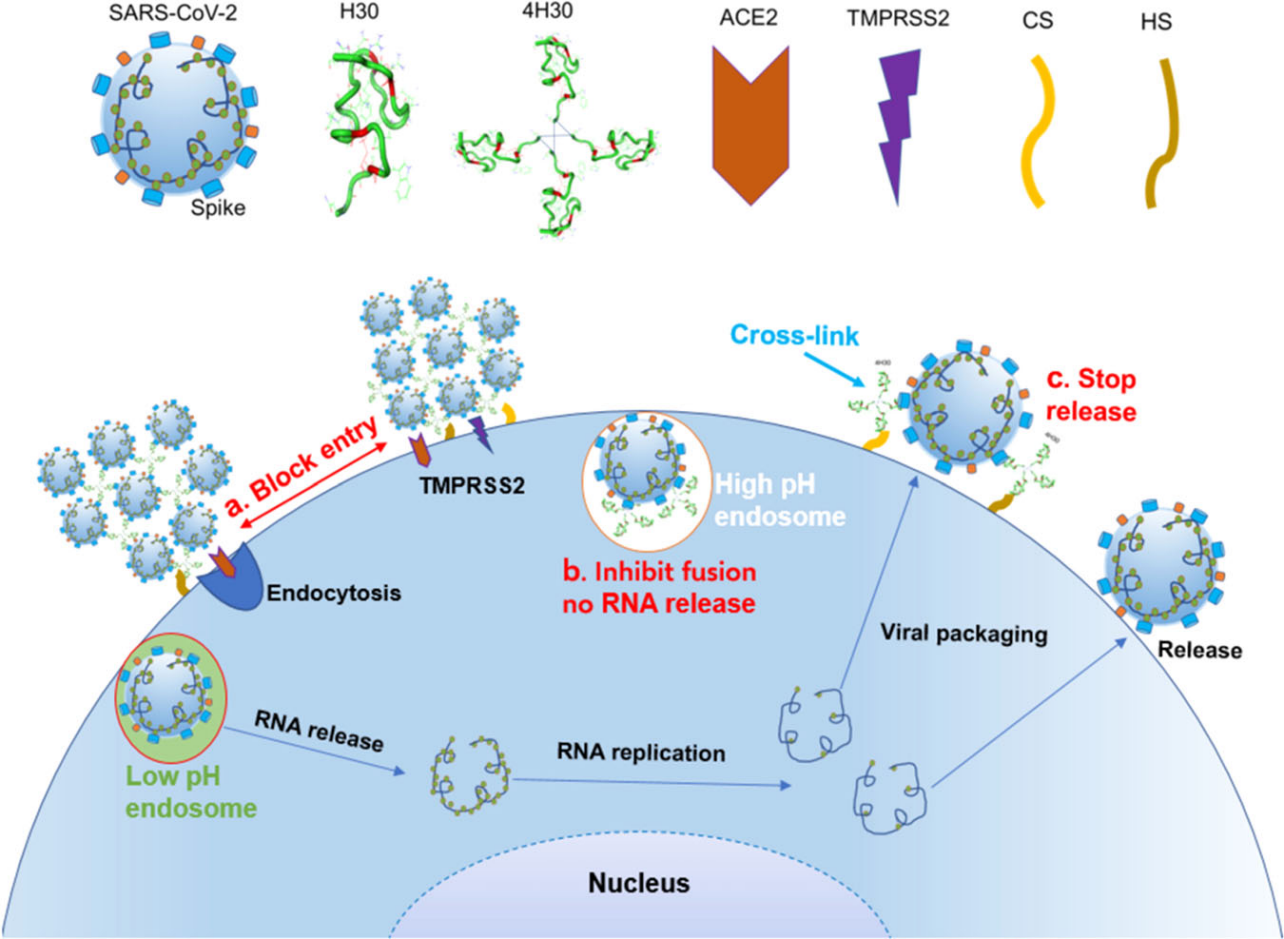
Schematic model of 4H30 inhibiting SARS-CoV-2 infection by triple mechanisms. **a** 4H30 can cross-link SARS-CoV-2 to form big virus clusters, which can block the two entry pathways in VeroE6 (mediated by endocytosis) and Calu-3 cells (mediated by TMPRSS2). **b** SARS-CoV-2, which is not cross-linked by 4H30 and enters cells by endocytosis, can be stopped in endosomes due to the inhibition of 4H30 on endosomal acidification, which blocks virus envelope fusion with the endosomal membrane. **c** 4H30 can stop viral release by cross-linking virus with cell-surface HS/CS. Collectively, 4H30 can inhibit viral entry, fusion, and release of SARS-CoV-2.

In this study, we provided a proof of concept that a branched basic peptide 4H30 could show triple antiviral functions against SARS-CoV-2 by blocking viral entry, fusion, and release (Fig. 6). 4H30 could provide a layer of protection on the cell surface to block both viral entry and release so as to suppress viral infection and dissemination. Notably, 4H30 could broadly inhibit SARS-CoV-2 variants with reduced syncytia in infected hamster lungs and more effectively inhibit viral replication of the Omicron variant because its replication is largely dependent on the endocytic pathway specially targeted by 4H30.

With the circulation of pandemic SARS-CoV-2, many variants with the potential to escape from neutralizing antibodies induced by vaccines are now important concerns. There is an urgency to find new antivirals with a broad range of activities against SARS-CoV-2. Currently, only a limited number of drugs have been approved for treating COVID-19 patients and are not widely available. Here, we identified a human-sourced defensin peptide 4H30 which contains the same segment of amino acid sequence as the original human beta-defensin 2. Thus, 4H30 is expected to be safe for clinical trials. Recent studies reported that human defensins could inhibit SARS-CoV-2^23,32,33^. However, the weak antiviral activity of defensins in the high salt condition and the difficulty to produce a sufficient quantity of recombinant HBD2 are major bottlenecks for manufacturers. In addition, the antiviral activity of HBD2 is poor^23^ and there is currently a paucity of in vivo data.

In this study, we demonstrated that 4H30 could broadly inhibit five SARS-CoV-2 strains with IC_50_ (< 0.89 μg/mL or 67 nM). 4H30 could bind to the SARS-CoV-2 spike and cross-link viral particles to block viral entry and basic 4H30 could inhibit endosomal acidification to block spike-ACE2 mediated fusion, similar to endosomal acidification inhibition of bafilomycin A1 and chloroquine to block spike-ACE2-mediated fusion^10^. The reduction of syncytia of 4H30-treated infectious lungs could be attributed to the inhibition of spike-ACE2-mediated cell fusion, which were found in patients with severe pneumonia^3^. Since spike protein expression on the infected cell surface can trigger the cell fusion with ACE2-expressed cells irrespective of whether a live virus is present, blocking cell-cell fusion by 4H30 or other fusion inhibitors might play an important role in reducing the syncytia formation and lung damage. Moreover, we demonstrated that cross-linking of viral proteins and cell surface GAGs could be a potential antiviral mechanism to affect viral attachment and inhibit viral release. Collectively, we have demonstrated a four-branched basic H30 (4H30), binding to spike and also GAGs, which effectively clusters viral particles on the cell membrane to block viral entry, fusion, and release of the newly packaged virus. Together with our previous 8P9R findings^10^, this kind of branched peptides might be an effective method^34^ to make peptides with more potent antiviral activities.

We found that 4H30 inhibited the Omicron variant more effectively when compared with the Delta variant. This is likely because the Omicron variant mainly uses the endocytic pathway but not the TMPRSS2 pathway^30,35^. The Omicron variant caused less body weight loss without syncytia formation in infected hamster lungs, which implicated that the Omicron variant might be attenuated when compared with Delta variant because TMPRSS2 is highly expressed in alveolar type I and type II cells of the lung^36^. The fusogenic and entry-pathway differences between variants might cause different sensitivity to drugs and clinical manifestations which require different treatments.

4H30 has shown potent antiviral activity against SARS-CoV-2 variants in hamsters with a low dose (0.5 mg/kg in hamsters), which may translate into a promising antiviral. 4H30 has triple antiviral mechanisms (clustering viral particles, blocking virus-cell fusion, and inhibiting viral release). It is likely that upcoming variants will remain susceptible to 4H30 and this study provides the proof of concept that a single antiviral can have multiple antiviral mechanisms as its antiviral strategy.

## Materials and methods

### Cell and virus cultures

Madin Darby canine kidney (MDCK, CCL-34), 293T (CRL-3216), A549 (CCL-185), VeroE6 (CRL-1586), VeroE6-TMPRSS2 (VeroE6-T), Calu-3 (HTB-55)^10^ cells from ATCC were cultured in DMEM or DMEM-F12K supplemented with 10% fetal bovine serum (FBS), 100 IU/mL penicillin and 100 μg/mL streptomycin. The virus strains used in this study included SARS-CoV-2 variants and MERS-CoV^11,13,37^, which were cultured in VeroE6 or VeroE6-T cells and viral titers were determined by plaque assay.

### Synthesis of peptides

According to our previous studies (30 amino acid P9 and P9R) related to mouse beta-defensin 4 (the ortholog of HBD2)^10–12^, we designed this 30 amino acid peptide H30 based on HBD2 and enhanced its antiviral activity by making the branched 4H30. H30, 2H30, 4H30, H26, and H23 shown in Fig. 1a were synthesized by ChinaPeptide (Shanghai, China). All peptides were dissolved in water. The solubility of peptides in water is greater than 5 mg/mL. The purity of all peptides was > 80%. The purity and mass of each peptide was verified by HPLC and mass spectrometry.

### Plaque reduction assay

Antiviral activity of peptides was measured by plaque reduction assay^38^. Briefly, peptides were dissolved in 150 mM PBS or 30 mM PBS (PBS/5). Peptides or bovine serum albumin (BSA, 0.2–25.0 μg/mL) were premixed with SARS-CoV-2 at room temperature. After 45–60 min of incubation, the peptide-virus mixture was transferred to VeroE6 cells for infection. At 1 hpi, infectious media were removed and 1% low melting agar was added to cells. Cells were fixed using 4% formalin at 2–3 dpi. Crystal blue (0.1%) was added for staining and the number of plaque was counted.

### Cytotoxicity assay

Cytotoxicity of peptides was determined by the detection of 50% cytotoxic concentration (CC_50_) using a tetrazolium-based colorimetric MTT assay^12^. Briefly, cells were seeded in a 96-well cell culture plate at an initial density of 4 × 10^4^ cells per well in DMEM supplemented with 10% FBS and incubated overnight. Cell culture media were removed and then DMEM supplemented with various concentrations of peptides and 1% FBS were added to each well. After 24 h incubation at 37 °C, MTT solution (5 mg/mL, 10 μL per well) was added to each well for incubation at 37 °C for 4 h. Then, 100 μL of 10% SDS in 0.01 M HCl was added to each well. After further incubation at room temperature with shaking overnight, the plates were read at OD_570_ using VictorTM X3 Multilabel Reader (PerkinElmer, USA). Cell culture wells without peptides were used as the experiment control and the medium only served as a blank control.

### Transmission electron microscopy assay

To determine the effect of 4H30 on viral particles, SARS-CoV-2 was pretreated with 50 μg/mL of 4H30 or H30 for 1 h. The virus was fixed by formalin overnight. For spike treated by 4H30 or H30, spike (50 μg/mL) mixed with 50 μg/mL of 4H30 or H30. Virus or protein samples were applied to continuous carbon grids. The grids were transferred into 4% uranyl acetate and incubated for 1 min. After removing the solution, the grids were air-dried at room temperature. Images were taken by FEI Tecnal G2-20 TEM or Philips CM100-TEM.

### ELISA assay

For ELISA assay^38^, peptides (1.0 μg per well) dissolved in H_2_O, ACE2 (100 ng), and chondroitin sulfate (CS, Sigma, Cat^#^ 230699, 300 ng) or heparan sulfate (HS, Sigma, Cat^#^ H7640, 300 ng) dissolved in PBS were coated onto ELISA plates and incubated at 4 °C overnight and was blocked at 4 °C overnight. Spike or ACE2 was added to 4H30 wells for binding and then determined by incubation with rabbit anti-spike (Sino, Cat^#^ 40590-T62, 1:8000) or rabbit anti-ACE2 (Takara, Cat^#^ A4612, 1:6000), or anti-His-HRP (Invitrogen, Cat^#^ R93125, 1:2000) at 37 °C for 30 min. 4H30 was added to CS or HS wells for binding and then determined by incubation with rabbit anti-HBD2 (Life Technologies, Cat^#^ PA5-103126). For determining spike and ACE2 binding, 4H30 or PBST was added to ACE2 for binding and then the spike was added to ACE2 for further incubation at 37 °C for 30 min. The binding ability of spike to ACE2 was determined by incubation with rabbit anti-spike (Sino, Cat^#^ 40590-T62, 1:8000) at 37 °C for 30 min and then incubation with goat-anti-rabbit HRP (Life Technologies, Cat^#^656120, 1:4000) at 37 °C for 30 min. The reaction was developed by adding 100 μL of TMB single solution (Life Technologies, Cat^#^ 002023) for 15 min at 37 °C and stopped with 50 μL of 1 M H_2_SO_4_. Readings were obtained in an ELISA plate reader (Victor 1420 Multilabel Counter; PerkinElmer) at 450 nm.

### Viral RNA extraction and quantitative reverse transcription PCR (RT-qPCR)

Viral RNA was extracted by Viral RNA Mini Kit (QIAGEN, Cat^#^ 52906, USA) according to the manufacturer’s instructions. Real-time RT-qPCR was performed as we described previously^38^. Extracted RNA was reverse transcribed to cDNA using PrimeScript II first Strand cDNA synthesis Kit (Takara, Cat^#^ RR036A) using GeneAmp^®^ PCR system 9700 (Applied Biosystems, USA). The cDNA was then amplified using specific primers (Supplementary Table S1) for detecting SARS-CoV-2 using LightCycle^®^ 480 SYBR Green I Master (Roach, USA). For quantitation, tenfold serial dilutions of standard plasmid equivalent to 10^1^ to 10^6^ copies per reaction were prepared to generate the calibration curve. The qPCR experiments were performed using LightCycler^®^ 96 system (Roche, USA).

### Endosomal acidification assay

Endosomal acidification was detected with a pH-sensitive dye (pHrodo green dextran, Invitrogen, Cat^#^ P35365) according to the manufacturer’s instructions as previously described but with slight modification^38^. First, VeroE6 cells were treated with DMEM, 4H30 (25.0 μg/mL), or bafilomycin A1 (50.0 nM) at 4 °C for 15 min. Second, VeroE6 cells were added with 100 μg/mL of pH-sensitive dye and DAPI and then incubated at 4 °C for 15 min. Before taking images, cells were further incubated at 37 °C for 15 min and then cells were washed twice with PBS. Finally, PBS was added to cells and images were taken immediately with a confocal microscope (Carl Zeiss LSM 800, Germany).

### Nucleocapsid (NP) immunofluorescence assay

According to our previous experiment^38^, VeroE6 cells were seeded on cell culture slides and were infected with SARS-CoV-2 (MOI = 0.01) and pretreated with DMEM or 4H30 (50.0 μg/mL). After 18 h infection, cells were fixed with 4% formalin for 1 h and then permeabilized with 0.2 % Triton X-100 in PBS for 5 min. Cells were washed by PBS and then blocked by 5% BSA at room temperature for 1 h. Cells were incubated with rabbit IgG anti-NP^25^ (1:4000) at room temperature for 1 h and then washed by PBS for next incubation with goat-anti-rabbit IgG Alexa-488 (Life Technologies, Cat^#^ A32731, 1:600) at room temperature for 1 h. Finally, cells were washed by PBS and stained with DAPI. Images were taken by confocal microscope (Carl Zeiss LSM 800, Germany).

### Viral entry fluorescence assay

To identify the effect of 4H30 on a virus, SARS-CoV-2 was pre-labeled with green Dio dye (Invitrogen, Cat^#^ 3898) according to the manufacturer’s instructions. The dio-labeled virus was treated by DMEM, 4H30, or H30 (25 μg/mL) for 45 min. VeroE6 or Calu-3 cells were infected by the pretreated virus for 1 h. For cells pretreated by 4H30, cells were pretreated by 4H30 for 1 h and then infected with a Dio-labeled virus. Infected cells were fixed by 4% formalin. The cell membrane was stained by membrane dye Alexa 594 (red, Invitrogen, W11262) and cell nuclei were stained by DAPI (blue). Virus entry or without an entry on the cell membrane was determined by confocal microscope (Carl Zeiss LSM 800, Germany).

### Viral release assay

SARS-CoV-2 (MOI = 0.01) was used to infect VeroE6 cells. At 6 hpi or 14 hpi, viral culture supernatants were removed. DMEM with or without 4H30 (50.0 μg/mL), BSA, CS, HS (10 μg/mL) was added to infected cells. At 10 hpi or 18 hpi, supernatants were collected to measure viral titers by RT-qPCR or fixed for anti-Spike immunofluorescence assay. After fixing at room temperature for 1 h, cells were blocked by 5% BSA for 1 h. Rabbit anti-spike (Sino, Cat# 40590-T62, 1:6000) and goat-anti-rabbit IgG Alexa-488 (Life Technologies, Cat^#^ A32731, 1:600) were incubated with cells at room temperature for 45 min. Images were taken by confocal microscope (Carl Zeiss LSM 800, Germany).

### ChABC and Hase treatment for viral release assay

VeroE6 cells were infected with SARS-CoV-2 (MOI = 0.1) at 37 °C and then treated by ChABC (sigma, Cat^#^ C2905, 1 U/mL), Hase I-III (Sigma, Cat^#^ H3917, 100 mU/mL) and Hase II (Sigma, Cat^#^ H6515, 100 mU/mL) at 14 hpi in the buffer according to the manufacture introduction. After 2 h incubation and washing of cells by PBS, DMEM with or without 4H30 (50 μg/mL) was added to cells for virus culture with the presence of ChABC and Hase. At 20 hpi, viral titers in cell supernatants were measured by RT-qPCR.

### Viral attachment, ChABC, and Hase digestion assay

VeroE6, A549, and Calu-3 cells were treated by DMEM, 4H30 (12.5 μg/mL), BSA, CS, HS (5 μg/mL), 4H30 + BSA, 4H30 + CS, or 4H30 + HS at 37 °C for 30 min and then washed by PBS. SARS-CoV-2 (MOI = 0.2) was added to cells for attachment at 4 °C for 1 h. After washing the unbound virus, the attached virus was measured by RT-qPCR. For ChABC and Hase assay, A549 cells were treated by ChABC (Sigma, Cat^#^ C2905, 1 U/mL), Hase I-III (Sigma, Cat^#^ H3917, 100 mU/mL), and Hase II (Sigma, Cat^#^ H6515, 100 mU/mL) at 37 °C for 2 h in the buffer according to the manufacture introduction. After washing cells with PBS, DMEM or 4H30 (12.5 μg/mL) was added to cells for 30 min incubation and then cells were washed with PBS. SARS-CoV-2 (MOI = 0.2) was added to cells for attachment at 4 °C for 1 h. The unbound virus was washed by PBS. The attached virus was measured by RT-qPCR.

### Spike-ACE2-mediated cell fusion assay

According to a previous study^10^, the pSpike of SARS-CoV-2, pACE2-human, or pGFP were transfected to 293T cells for protein expression. After 24 h, to trigger the spike-ACE2-mediated cell fusion, 293T-Spike-GFP cells were cocultured with 293T-ACE2 or Calu-3 cells with the supplement of drugs (4H30, bafilomycin A1, or camostat). The 293T-GFP cells were cocultured with 293T-ACE2 or Calu-3 cells as the negative control. After 6–8 h of co-culture, five fields were randomly selected in each well to take the cell fusion pictures by fluorescence microscopes.

### Western blot assay

Spike, ACE2 (7.5 μg) or PBS was premixed with 4H30 (10 μg) and then was passed through 30 kDa centrifugal filters to elute and wash the unbound 4H30 for more than 200-folds. The remained proteins in the centrifugal filters were loaded to 15% PAGE. 4H30 was detected by rabbit anti-HBD2 (Thermo Scientific, Cat^#^ PA5-103126, 1:5000) and goat-anti-rabbit HRP as a secondary antibody (Thermo Scientific, 656120, 1:4000 dilution).

### Antiviral assay in animals

Female hamsters (6–8-week old)^27^ were kept in biosafety level 2/3 laboratory (housing temperature between 22–25 °C with dark/light cycle) and given access to standard pellet feed and water ad libitum. All experimental protocols followed the standard operating procedures of the approved biosafety level 2/3 animal facilities. Animal ethical regulations were approved by the Committee on the Use of Live Animals in Teaching and Research of the University of Hong Kong^39^. To evaluate the drug toxicity in vivo, hamsters and mice were intranasally inoculated with 4H30 (0.5 mg/kg). Two more doses were given to hamsters or mice on the following day. Lung tissues were harvested at day 4 for Haemotoxylin and Eosin (H&E) staining. To evaluate the pre-exposure antiviral activity, 4H30, H23, or PBS was intranasally inoculated to hamsters at 24, 8, and 4 h or 2 min (pre) before SARS-CoV-2 (B.1.1.63) inoculation (500 PFU). Lung tissues were collected at 2 dpi for determining viral titers and histopathology. To evaluate the post-exposure antiviral activity, hamsters were intranasally inoculated with SARS-CoV-2 (B.1.1.63, Delta, or Omicron, 250 PFU). At 8 hpi, PBS, 4H30, camostat (1 mg/kg) or remdesivir (0.5 mg/kg) was intranasally given to hamsters. Two more doses were given to hamsters on the following day for sample collection at 2 dpi and four more doses were given to hamsters in the following 2 days for sample collection at 3 dpi. Viral loads in hamster lungs or nasal wash were measured by plaque assay and histopathology were measured by H&E staining at 2 dpi or 3 dpi.

### Statistical analysis

The statistical significance of the results was calculated by the two-tailed Student’s *t*-test. Results were considered significant at *P* < 0.05.

## Supplementary information

Supplementary Information

## Data Availability

All data that support the conclusions of the study are available from the corresponding author upon reasonable request.
