# Supplementary material for: A trifunctional peptide broadly inhibits SARS-CoV-2 Delta and Omicron variants in hamsters
Source: Cell Discov. 2022 Jun 30;8:62. doi: 10.1038/s41421-022-00428-9 (PMC9243000; doi:10.1038/s41421-022-00428-9)
Supplement: Supplementary file 1 — Supplementary Information [file 41421_2022_428_MOESM1_ESM.pdf]

Supplementary information

A triple-functional peptide broadly inhibits SARS-CoV-2 Delta and Omicron variants in hamsters

Hanjun Zhao, Kelvin K. W. To, Hoiyan Lam, Chuyuan Zhang, Zheng Peng, Xinjie Meng, Xiankun Wang, Anna, Jinxia Zhang, Bingpeng Yan, Jianpiao Cai, Man Lung Yeung, Jasper Fuk-Woo Chan, Kwok-Yung Yuen

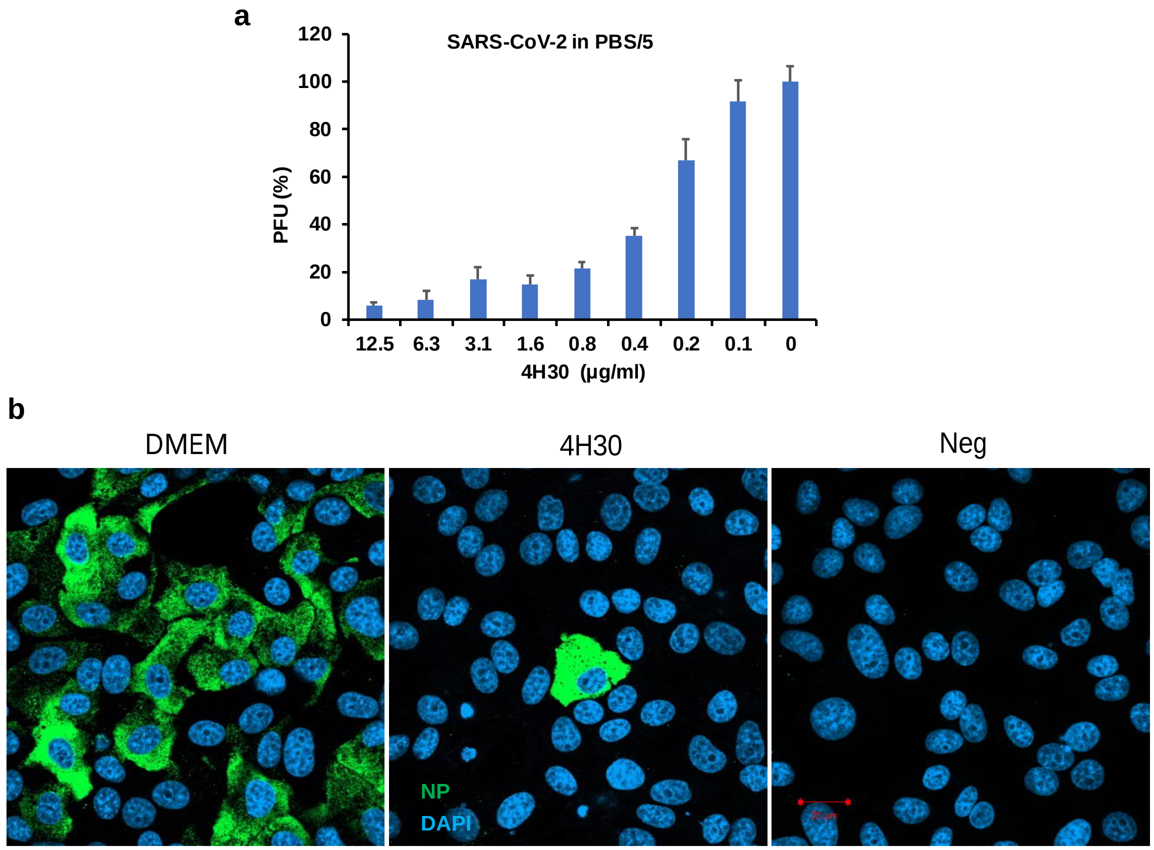

Supplementary Fig. S1. 4H30 significantly inhibited viral replication in VeroE6 cells. (**a**) 4H30 inhibited viral infection in the low salt condition (*n* = 4). SARS-CoV-2 was mixed with indicated concentration of 4H30 in the low salt condition (PBS/5) for plaque reduction assay. Data are presented as mean ± SD of four independent biological samples. (**b**) 4H30 inhibited viral replication determined by anti-nucleocapsid immunofluorescent staining. SARS-CoV-2 (1000 PFU) with or without 4H30 (50 μg/mL) treatment was added to VeroE6 cells for infection. Cells were fixed at 18 hpi and anti-nucleocapsid immunofluorescent staining was used to show the viral replication. Cell only was the negative control (Neg). Scale bar = 20 μm. Representative images were taken by confocal microscope. Experiments were repeated twice independently.

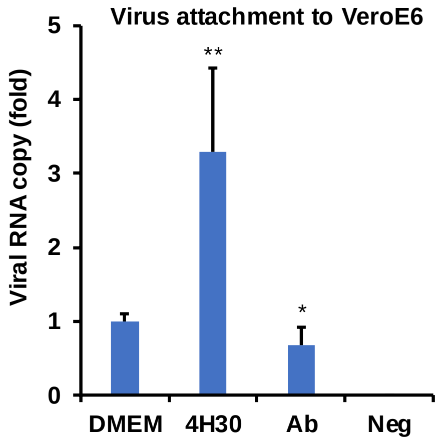

Supplementary Fig. S2. 4H30 increased the attachment of SARS-CoV-2 on VeroE6 cells. SARS-CoV-2 was pre-mixed with 4H30 (25 μg/mL) or neutralizing antibody (Ab) for 1 hour, and then was added to VeroE6 cells for attachment at 4℃ for 1 hour (n=4). Viral RNA copies in cell lysate were measured by RT-qPCR after washing the non-attached virus. Cells without infection were the negative control (Neg). Viral RNA copy (fold) was normalized to that of DMEM * indicates $P < 0.05$. ** indicates the $P < 0.01$ when compared with DMEM. *P* values were calculated by the two-tailed Student's t test. Data are presented as mean ± SD of four independent biological samples.

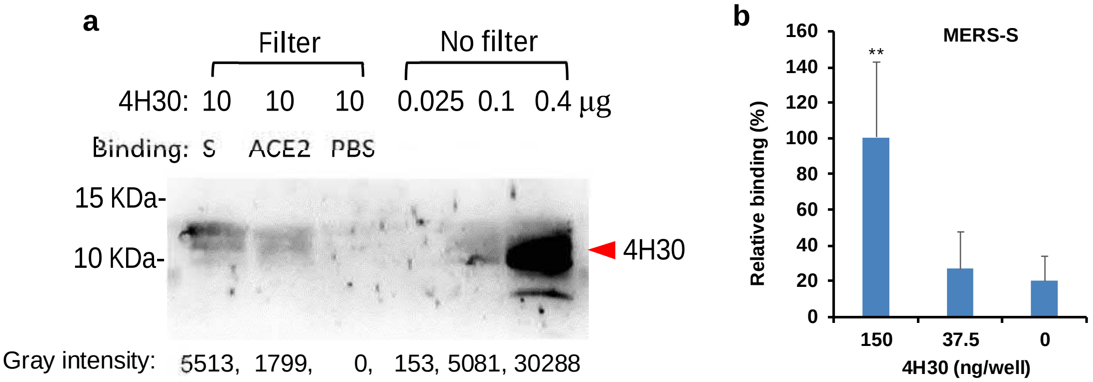

Supplementary Fig. S3. 4H30 could effectively bind to spike protein. (**a**) Binding to spike of SARS-CoV-2. Spike (S, 7.5 µg) or ACE2 (7.5 µg) or PBS was mixed with 4H30 (10 µg) for 1 hour and then the mixture was parallelly passed through three centrifugal filters (30 kDa) to elute the unbound 4H30 (12 kDa) by >200-fold elution. The 4H30 binding to spike (170 kDa) or ACE2 (70 kDa), which was remained in the column (30 kDa), was determined by anti-H30 staining in the same experiment. 4H30 (0.025, 0.1, 0.4 µg) without passing the centrifuge filter was used as the positive control. Results indicated that 4H30 could more effectively bind to spike when compared with ACE2. 4H30 in PBS group was not detectable. (**b**) 4H30 binding to spike of MERS-CoV ($n = 5$). Indicated 4H30 was coated on ELISA plate and blocked by 2% BSA. Spike of MERS-CoV (200 ng) was added to the wells for binding to 4H30. Relative OD_{450} was normalized to that of 4H30 (150 ng). ** indicates $P < 0.01$ when compared with the negative control (0). P values were calculated by the two-tailed Student's t test. Data are presented as mean ± SD of five independent biological samples.

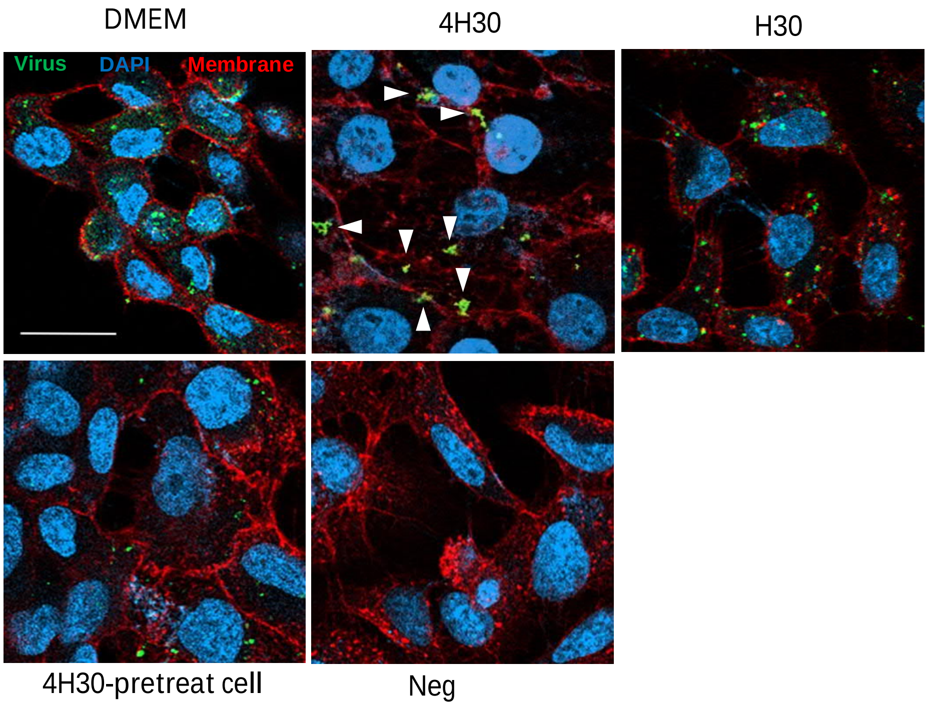

Supplementary Fig. S4. 4H30 could cluster SARS-CoV-2 on the membrane of VeroE6 cells. SARS-CoV-2 (B.1.1.63) was pre-labelled by green fluorescence dye and then treated by DMEM, 4H30, or H30 (25 μg/mL) before infecting cells or cells were pretreated by 4H30 (4H30-pretreat cell) before virus infection. After 1 h infection, cells were fixed and stained by cell membrane dye (red) and nuclear dye (blue). Cells without infection were the negative control (Neg). White triangles indicated the big clustered viral particles located at cell membrane. Scale bar = 20 μm. Experiments were repeated twice independently.

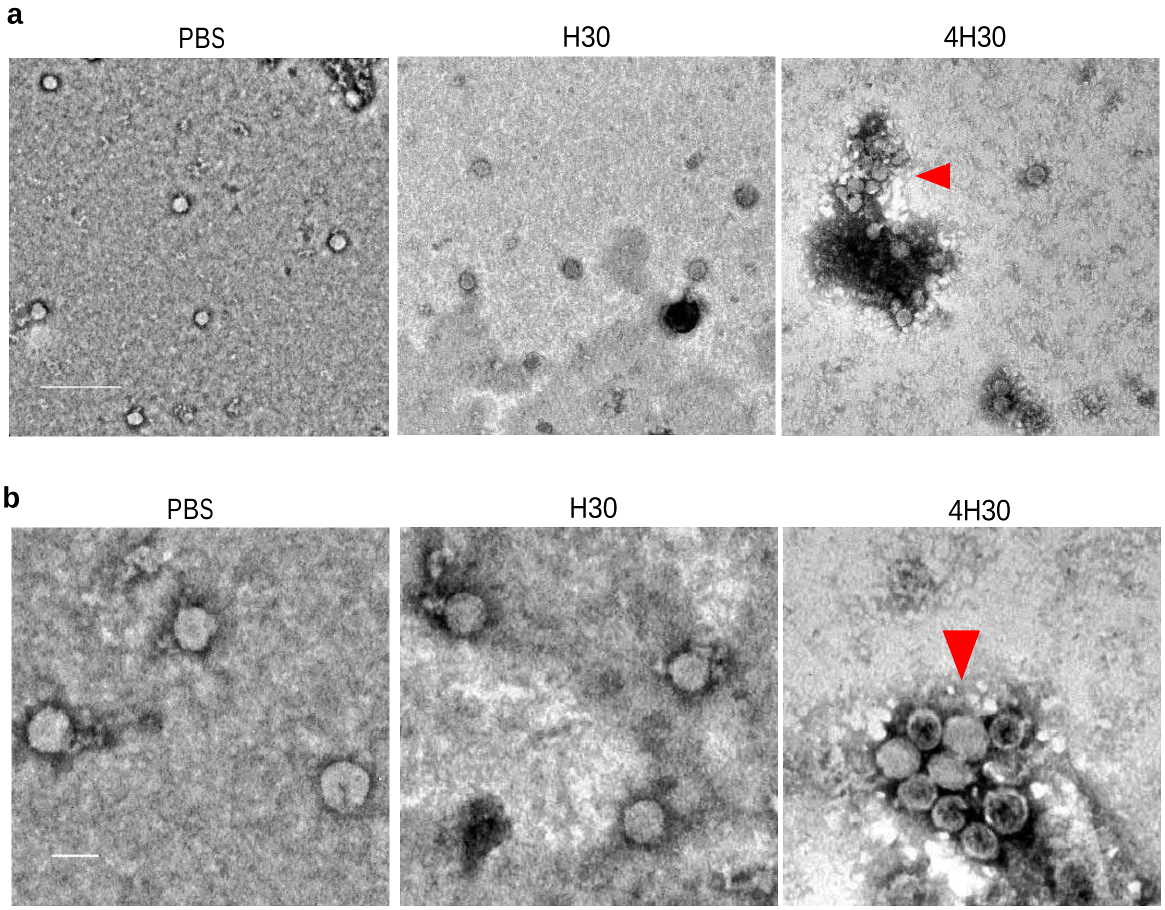

Supplementary Fig. S5. 4H30 could cluster SARS-CoV-2 virus. SARS-CoV-2 was treated with PBS, H30, or 4H30 (50 μg/mL) for 1 hour. The treated virus was negatively stained for TEM assay. The red triangles indicated the big cluster of cross-linked SARS-CoV-2. Scale bar = 500 nm for (**a**) and scale bar =100 nm for (**b**). The big viral particles in 4H30-treated samples were more than 400 nm, which was bigger than the size (~100 nm) of the normal SARS-CoV-2 virion. Representative images were taken by FEI Tecnai G2-20-TEM. Experiments were repeated twice independently. The (**a**) and (**b**) panels represented two different fields.

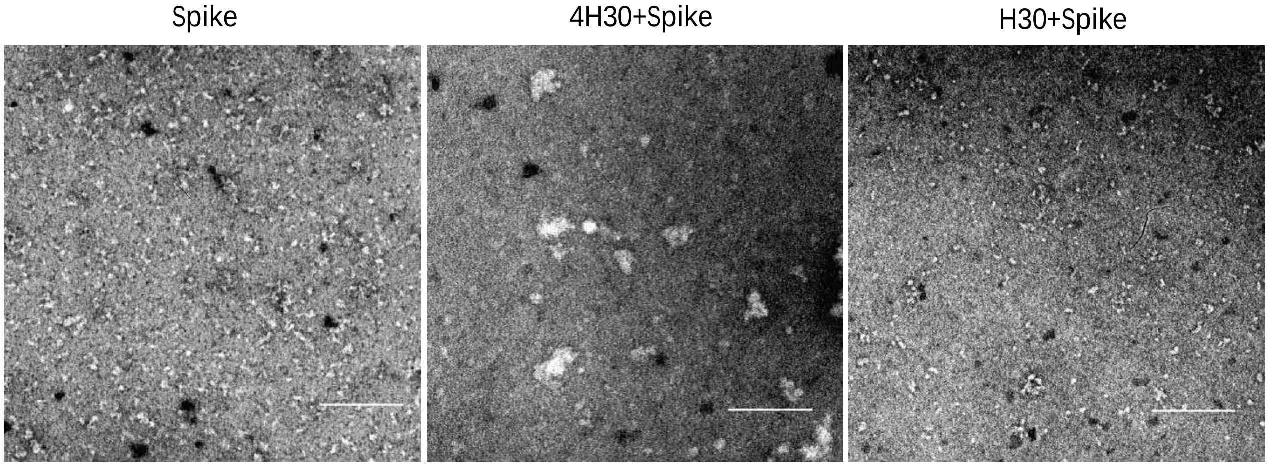

Supplementary Fig. S6. 4H30 could cluster spike protein. Spike protein (100 µg/mL) mixed with 100 µg/mL of 4H30 (spike+4H30) or H30 (spike+H30). Spike only was used as the control. The treated spike was negatively stained for TEM assay. The white particles in 4H30+Spike figure indicated the big cluster of cross-linked spike protein (> 50 nm). Scale bar = 200 nm. The normal spike protein was <10 nm. Representative images were taken by Philips CM100-TEM. Experiments were repeated twice independently.

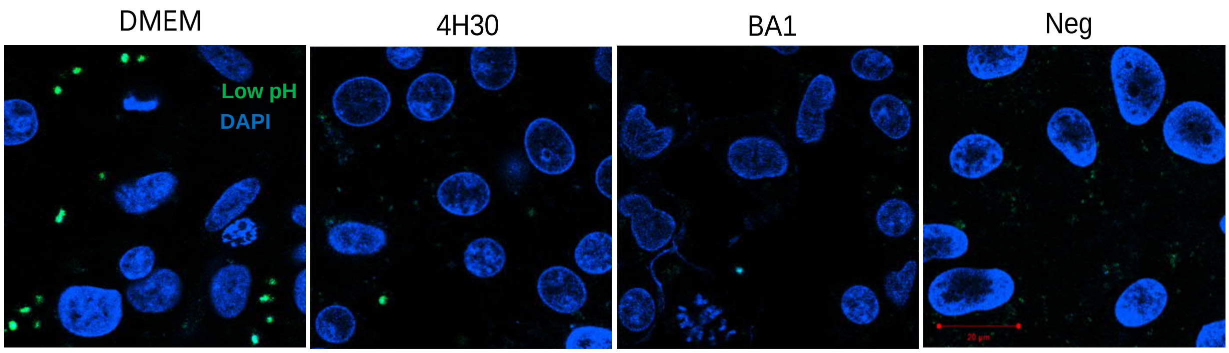

Supplementary Fig. S7. 4H30 could inhibit endosomal acidification in VeroE6 cells. VeroE6 cells were treated with pH-sensitive dye (green in low pH endosomes) and the indicated inhibitor 4H30 (25 µg/mL), bafilomycin A1 (BA1, 50 nM) or DMEM. Nuclei were stained as blue. Cell only without treatment was the negative control (Neg). Representative live-cell images were taken by confocal microscope. Experiments were repeated with two independent biological samples. Scale bar = 20 µm.

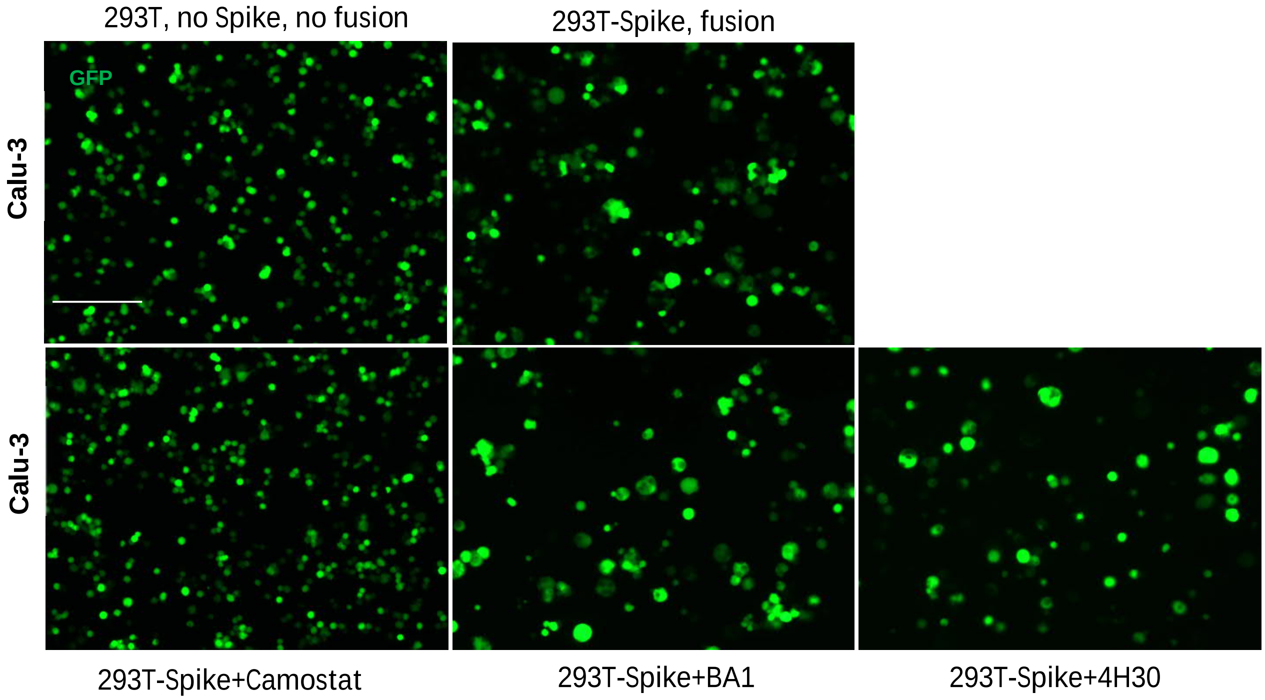

Supplementary Fig. S8. 4H30 could not inhibit spike-ACE2 mediated fusion between 293T-Spike and Calu-3 cells. Calu-3 cells were co-cultured with 293T or 293T-Spike with the treatment of inhibitor 4H30 (125 µg/mL), bafilomycin A1 (BA1, 50 nM), camostat (100 µg/mL). Cell 293T with the expression of spike (293T-Spike) and without spike (293T) were the fusion positive and negative control. Representative images were taken at 6-8 h post-coculture. Cell fusion sizes in cells of 293T-Spike, 293T-Spike-4H30, and 293T-Spike-BA1 were > 40 µm, which were bigger than the normal non-fusion cell size (~10 µm) of 293Tcells. Camostat could show the inhibition on cell fusion when compared with the size of 293T-Spike cells. Experiments were repeated twice independently. Scale bar =200 µm.

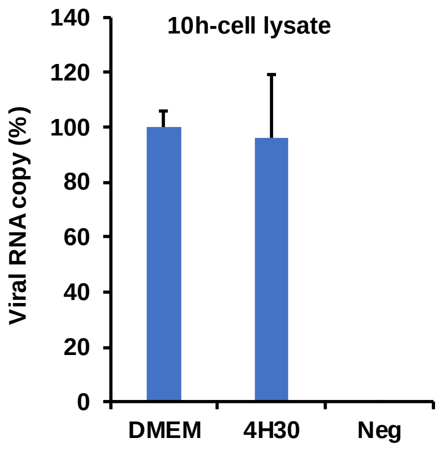

Supplementary Fig. S9. SARS-CoV-2 replication in Calu-3 cells treated by 4H30 at 6 hpi (*n* = 4). Peptide 4H30 (12.5 µg/mL) was added to cells at 6 hpi. Viral RNA copies in cell lysate were measured at 10 hpi. Cells without infection were the negative control (Neg). Viral RNA copy (%) was normalized to that of DMEM. Data are presented as mean ± SD of four independent biological samples.

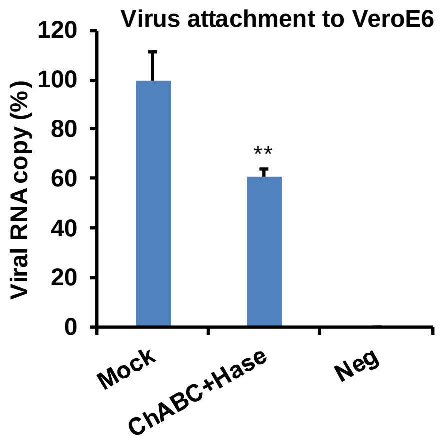

Supplementary Fig. S10. Cleaving cellular glycosaminoglycans could reduce SARS-CoV-2 attachment to VeroE6 cells. VeroE6 cells were treated by Chondroitinase ABC (ChABC) and Heparinase (Hase) to remove cell surface CS and HS and then SARS-CoV-2 was added to cells for attachment at 4 °C. Viral RNA copies were measured by RT-qPCR at 1 hpi. Cells without infection were the negative control (Neg). ** indicates $P < 0.01$ when compared with infected cells treated by buffer (Mock). *P* values were calculated by the two-tailed Student's *t* test. Data are presented as mean ± SD of four independent biological samples.

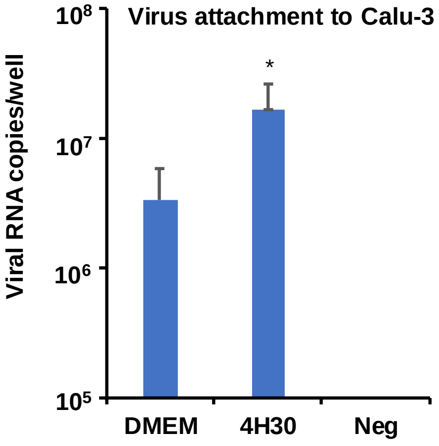

Supplementary Fig. S11. 4H30 could increase SARS-CoV-2 attachment to Calu-3 cells. Calu-3 cells were treated by 4H30 (12.5 μg/mL) and then SARS-CoV-2 was added to cells for attachment at 4 °C. Viral RNA copies were measured by RT-qPCR at 1 hpi. Cells without virus infection were the negative control (Neg) * indicates $P < 0.05$ when compared with DMEM. P value was calculated by the two-tailed Student's t test. Data are presented as mean ± SD of four independent biological samples.

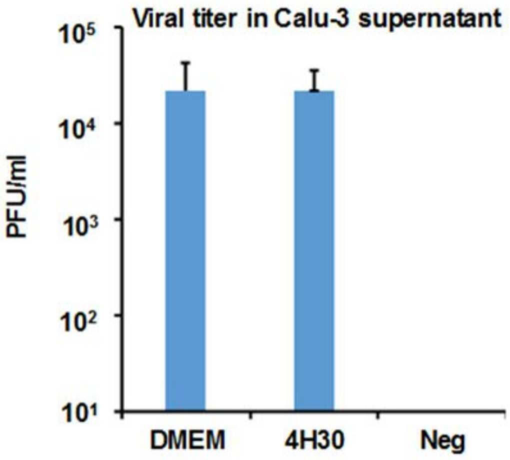

Supplementary Fig. S12. SARS-CoV-2 replication in Calu-3 cells. Calu-3 cells were pretreated by 4H30 (12.5 μg/mL) for 1 h and then 4H30 was removed and washed by PBS. SARS-CoV-2 was added to cells for infection. The viral titers in supernatants of cells treated with 4H30 or DMEM were measured at 24 hpi. Cells without infection were the negative control (Neg). Data are presented as mean ± SD of four independent biological samples.

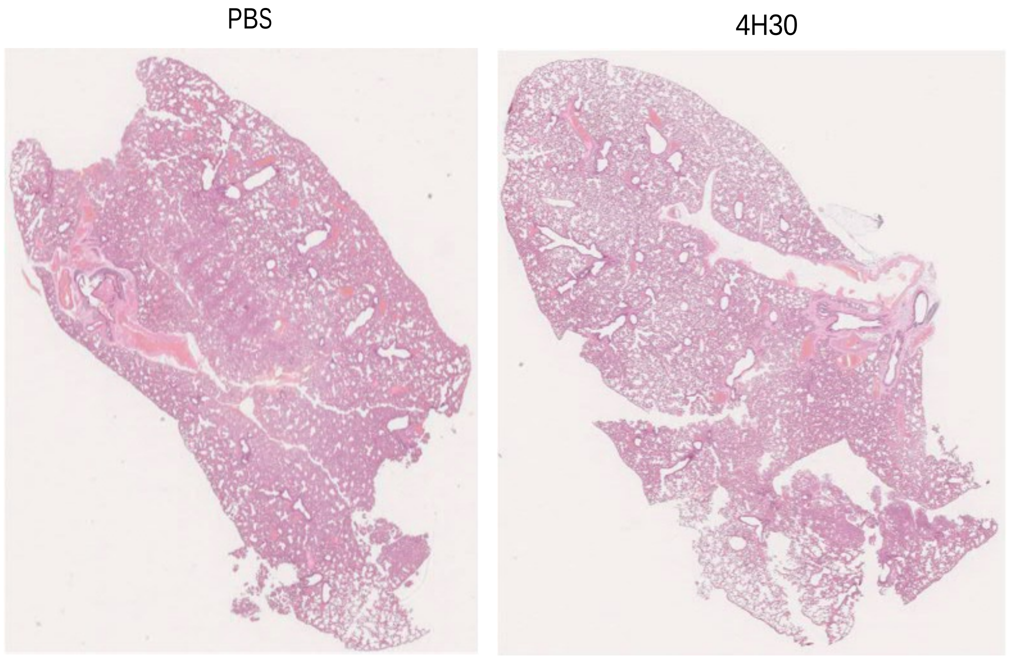

Supplementary Fig. S13. Full images of histopathology of hamster lungs. Hamsters were intranasally inoculated with PBS or 4H30. Three intranasal doses of 4H30 (0.5 mg/kg) were administered within two days. The lung tissues were harvested at day 4 post-inoculation for H&E staining and full images were taken by NanoZoomer Digital Pathology System.

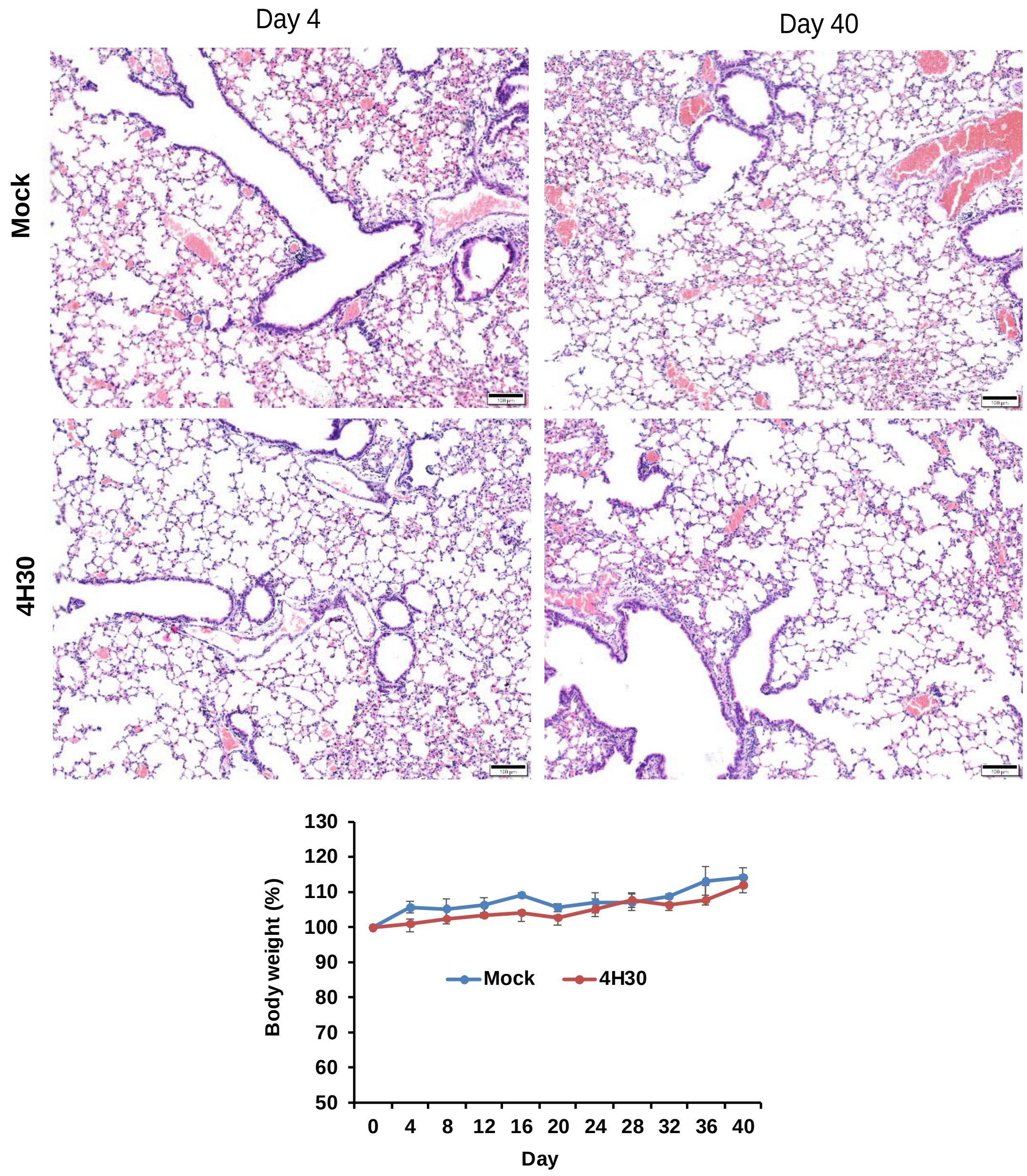

Supplementary Fig. S14. Histopathology and body weight changes of mice inoculated with 4H30. Mice were intranasally inoculated with 4H30 (0.5 mg/kg) with three doses withing two days. The lung tissues were collected at day 4 and day 40 after inoculation. Body weight was monitored for 40 days. PBS-treated mice (Mock) were included as the normal control. Scale bar = 100 μm. Data are presented as mean ± SD of three mice in each group.

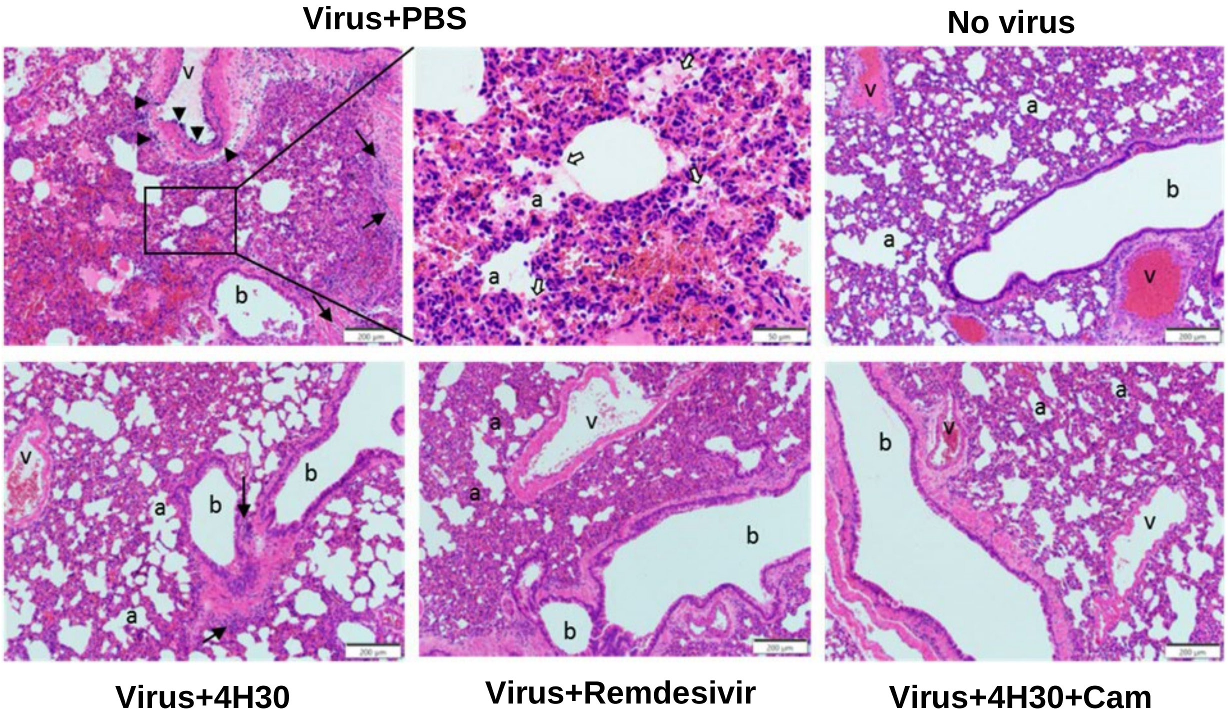

Supplementary Fig. S15. Histopathological assay of infected lungs. Hamsters were challenged with Delta variant and treated by PBS, 4H30, remdesivir or 4H30+Camostat. Lung tissues were harvested at 3 dpi. The images showed diffuse lung consolidation in virus-infected lungs treated by PBS. A bronchial section (b) showed that epithelium was damaged and detached in the lumen; immune cell infiltration was observed inside and round the bronchial wall (arrows). A big pulmonary blood vessel (v) showed severe immune cells infiltration (arrowheads). The squared area was magnified and showed alveolar wall destruction, alveolar space hemorrhage, mononuclear cell infiltration and exudation (a and open arrows). The lung treated with 4H30 had no apparent epithelium damage, only small foci of peri-bronchial wall immune cell infiltration (arrows). A pulmonary blood vessel (v) showed normal structure. Alveolar structure (a) showed no consolidation. The image of lung treated by remdesivir showed two sections of bronchial (b) and no peri-bronchial infiltration. Alveolar wall (a) thickening with increase immune cells, but no air space infiltration. The image of lung treated by 4H30 and Camostat (4H30+Cam) showed that lung tissues were largely normal with bronchial lining epithelial cells death (b), no alveolar structure (a) destruction, blood vessels (v) are normal. Hamster lung without virus infection was the normal control. Scale bar=200μm and 50μm, respectively.

Supplementary Table S1. Primers of RT-qPCR.

Gene	Primer	Oligonucleotide sequence (5' to3')
SARS-CoV-2	S-F	CCTACTAAATTAAATGATCTCTGCTTTACT
	S-R	CAAGCTATAACGCAGCCTGTA
